# Automated Analysis of Cryptococcal Macrophage Parasitism Using GFP-Tagged Cryptococci

**DOI:** 10.1371/journal.pone.0015968

**Published:** 2010-12-31

**Authors:** Kerstin Voelz, Simon A. Johnston, Julian C. Rutherford, Robin C. May

**Affiliations:** 1 School of Biosciences, University of Birmingham, Birmingham, United Kingdom; 2 Institute for Cell and Molecular Biosciences, Medical School, University of Newcastle, Newcastle upon Tyne, United Kingdom; University of Minnesota, United States of America

## Abstract

The human fungal pathogens *Cryptococcus neoformans* and *C. gattii* cause life-threatening infections of the central nervous system. One of the major characteristics of cryptococcal disease is the ability of the pathogen to parasitise upon phagocytic immune effector cells, a phenomenon that correlates strongly with virulence in rodent models of infection. Despite the importance of phagocyte/*Cryptococcus* interactions to disease progression, current methods for assaying virulence in the macrophage system are both time consuming and low throughput. Here, we introduce the first stable and fully characterised GFP–expressing derivatives of two widely used cryptococcal strains: *C. neoformans* serotype A type strain H99 and *C. gattii* serotype B type strain R265. Both strains show unaltered responses to environmental and host stress conditions and no deficiency in virulence in the macrophage model system. In addition, we report the development of a method to effectively and rapidly investigate macrophage parasitism by flow cytometry, a technique that preserves the accuracy of current approaches but offers a four-fold improvement in speed.

## Introduction

*Cryptococcus neoformans* (serotypes A and D) and *C. gattii* (serotypes B and C) are encapsulated basidiomycetous yeasts that are the causative agents of cryptococcosis [1]. *C. neoformans* mainly infects immunocompromised individuals for example those with HIV infection, leukemia and other cancers or undergoing corticosteroid treatment [2]. Within this species, serotype A isolates have been found to cause the majority of infections [2]. In contrast, *C. gattii* is a primary and emerging pathogen of healthy individuals; in particular in North America where serotype B subgroup VGIIa is responsible for approximately 95% of infections in outbreaks in British Columbia, Canada and in the Pacific Northwest [3], [4].

Cryptococcal infection can be asymptomatic, chronic or acute. Infection is thought to occur via inhalation of desiccated yeast cells or spores [1], [5]. The initial pulmonary colonization is most often asymptomatic or presents with cold-like symptoms such as coughing and mild fever but pneumonia and acute respiratory stress syndrome have been reported in severe cases [6], [7]. The disease then typically disseminates from the primary site of infection to the central nervous system leading to meningitis and meningoencephalitis that are fatal without rapid clinical intervention. In addition, cryptococcosis can occasionally present as a secondary infection of skin, lungs, prostate and eye [2]. Globally, cases of cryptococcal meningitis are estimated to be 957,900 of which 624,700 result in death each year [8].

Macrophages seem to play a critical role in the progression and outcome of cryptococcal infections. Yeast cells are internalised by alveolar macrophages shortly after infection [9], [10] and can then survive and proliferate within the host cell, eventually escaping by cell lysis [10], [11], [12], [13], [14], [15], [16] or a novel non-lytic expulsive mechanism [17], [18]. The ability to reside within macrophages, together with the phenomenon of lateral yeast cell transfer from one macrophage to another [11], [14] have led to the suggestion that cryptococci may disseminate within the host via a ‘Trojan Horse’ mechanism [19], [20].

Research into the interaction between *Cryptococcus* and macrophages in recent years has established this as a model system for investigation of cryptococcal virulence, since intracellular yeast proliferation in macrophages correlates well with virulence data from mice [15], [21]. However, experimental approaches to quantify macrophage parasitism rely on time-lapse imaging and/or manual colony counts of isolated yeast, both of which are low throughput, time-consuming methods. To facilitate faster analysis of cryptococcal intracellular parasitism, we have been investigating the use of fluorescently tagged yeast together with flow cytometry. Here, we report the production of two cryptococcal strains that show strong, stable GFP expression: a GFP-positive *C. neoformans* serotype A type strain H99 and a GFP-positive *C. gattii* serotype B type strain R265 (an isolate from the ongoing Vancouver Island Outbreak [4]). We have extensively characterised both strains and show that their responses to environmental and host stress conditions and their virulence in the macrophage model system remain unaltered. Using these strains, we report a flow cytometric approach for accurate, high throughput quantification of macrophage parasitism. Taken together, these findings represent a powerful resource for the cryptococcosis research community and should facilitate rapid advances in our understanding of cryptococcal virulence.

## Results

### Random genomic integration and expression of a GFP construct in *C. neoformans* H99 and *C. gattii* R265

**Figure 1:**
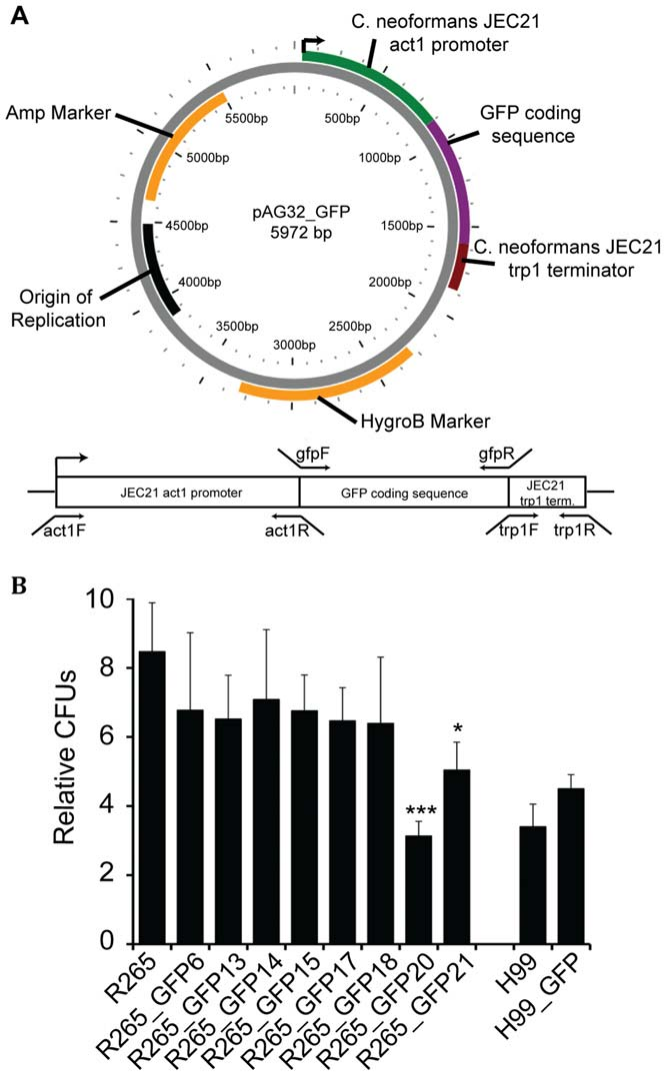
GFP expressing mutants. (A) Plasmid map of pAG32 containing GFP construct and GFP-construct with primer localisation below. (B) Growth rate analysis of the GFP-positive strains. Yeast were grown in YPD media at 25°C with rotation (20 rpm). Colony forming units were counted at time point 0 and 24 hours and relative strain growth calculated. Plasmid map was created using the PlasMapper 2.0 [60]. (* P-Value <0.05, *** P-Value <0.001).

To achieve constitutive GFP expression, we created a construct where GFP is under the control of the actin promoter and tryptophan terminator. Both sequences, obtained from NCBI for the *C. neoformans* strain JEC21 (AY483215), were used for the design of overlapping primers. The three overlapping PCR products were combined into the vector pRS426 [22] to create a GFP construct using the homologous recombination machinery of *S. cerevisiae*. Afterwards, the construct was recovered by PCR and ligated into the vector pAG32 [23], which was used as shuttle vector for biolistic bombardment (Figure 1A). Three individual biolistic transformations yielded a range of hygromycin B resistant and GFP expressing *C.* gattii R265 colonies but only two transformant *C. neoformans* H99 colonies. All positive colonies were re-streaked onto selective YPD plates. Eight *C. gattii* R265 strains (R265_GFP6, R265_GFP13, R265_GFP14, R265_GFP15, R265_GFP17, R265_GFP18, R265_GFP20, R265_GFP21) stably expressing GFP were randomly selected for further analysis. Of the two transformed H99 colonies, one showed unstable GFP expression that was lost after being re-streaked and hence only a single GFP-expressing H99 strain was used for further analysis. As a first step to analyse these GFP-strains, we assessed growth in YPD media at 25°C. Strains R265_GFP20 and R265_GFP21 showed a statistically significant reduction in growth rate in YPD compared to the parental R265 strain and these two strains were therefore excluded from further analysis. However, none of the remaining mutants were altered in their ability to growth in YPD media (Figure 1B).

### GFP expressing strains show no altered response to stress conditions

**Figure 2:**
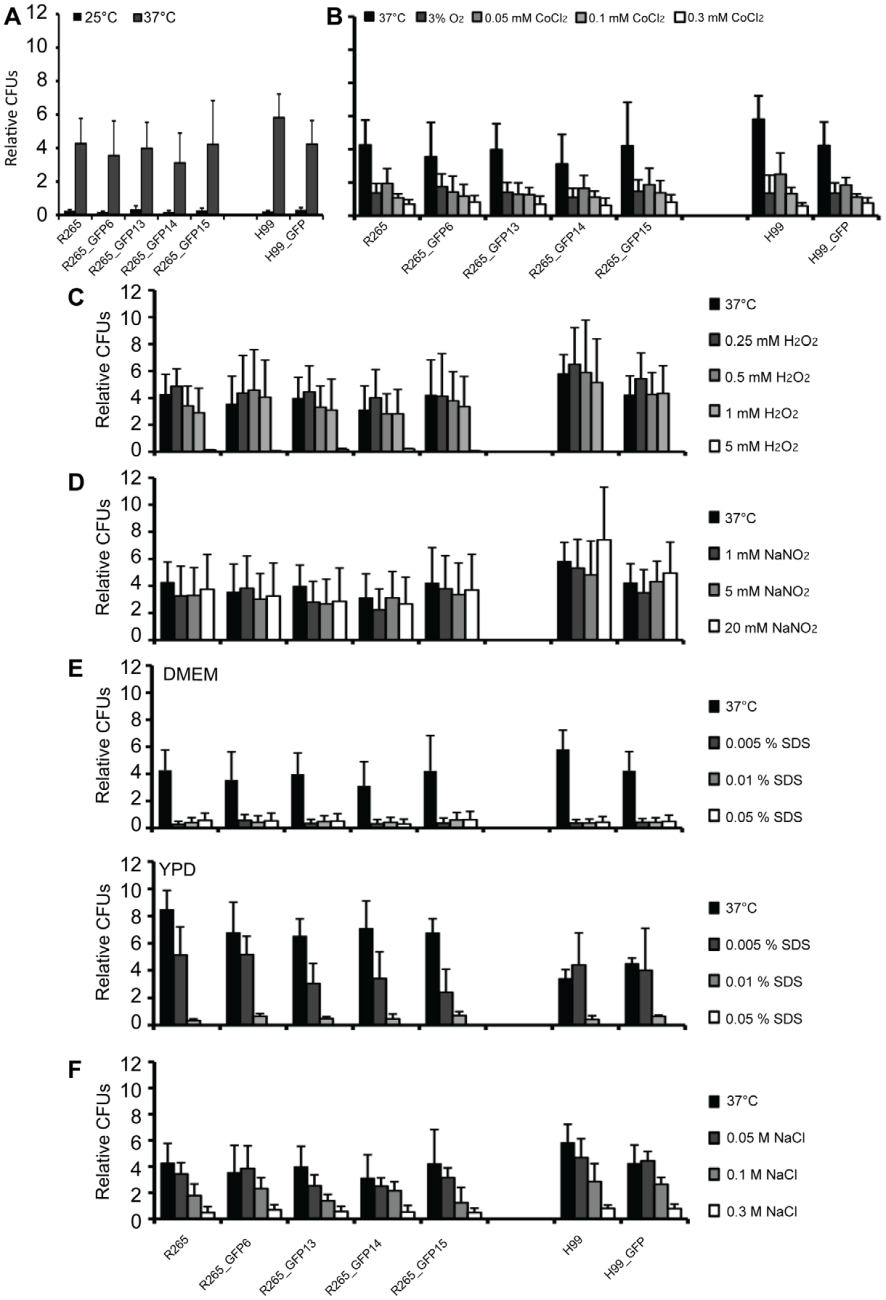
Response of GFP expressing strains to stress. The stress tolerance of the GFP-expressing isolates was tested by incubation under stress conditions for 24 hours followed by relative CFU analysis. Neither high temperature (A), hypoxia (B), oxidative stress (C), nitrosative stress (D), SDS exposure (E) or high salt (F) resulted in any significant differences in survival relative to non-GFP expressing parental strains (Table S1). Data are presented as means of at least three independent repeats with 2 times standard error.

Within this host environment *Cryptococcus* encounters a variety of stresses. The five selected GFP-positive strains were tested in an *in vitro* system for their ability to cope with different stress conditions in comparison with their parental strains (Figure 2 and Table S1). Growth rates of all five GFP-expressing strains were indistinguishable from their respective parental strains at two different temperatures (25°C and 37°C). In addition, none of the strains were altered in their response to hypoxia (3% oxygen or treatment with 0.05, 0.1 and 0.3 mM CoCl_2_ [24]), oxidative (0.25, 0.5, 1 and 5 mM H_2_O_2_) or nitrosative (1, 5 and 20 mM NaNO_2_) stress, or exposure to cell wall damage (0.005, 0.01 and 0.05% SDS or 0.05, 0.1 and 0.3 M NaCl). NaNO_2_ treatment did not reduce growth at any of the chosen concentrations. However, concentrations as low as 250 µM and 500 µM NaNO_2_ have been shown to induce a transcriptional or translational response, respectively, to this stress [25]. Hence, we believe the chosen concentrations were sufficient to reveal potential stress-mediated growth defects due to integration of GFP. Thus, none of the GFP-expressing strains show any significant changes in their response to stress conditions.

### GFP expressing strains show no alteration in virulence in macrophages

**Figure 3:**
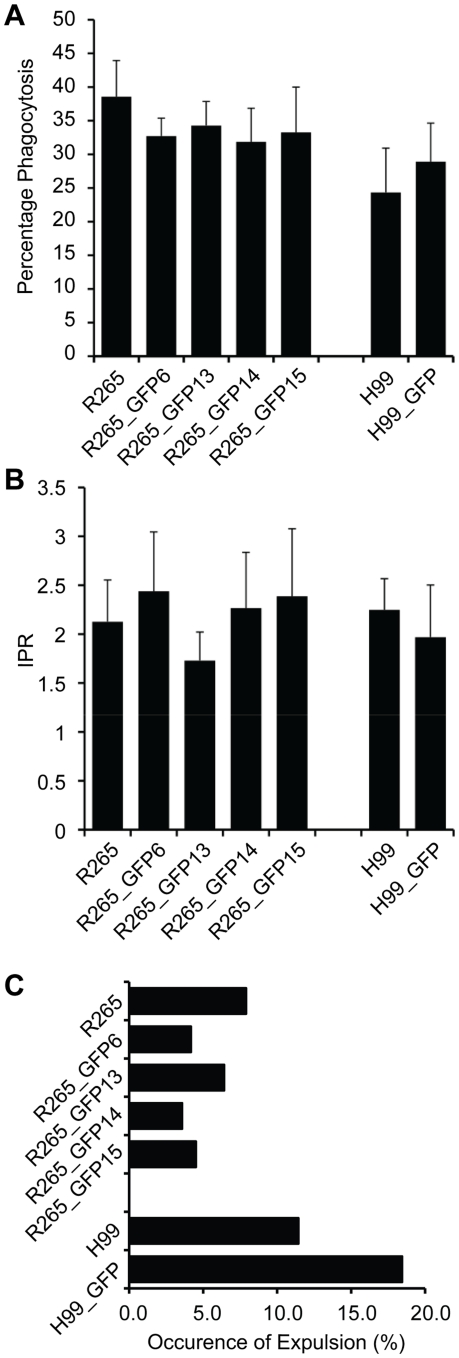
Analysis of intracellular virulence parameters. J774 macrophages were infected with GFP-positive isolates or their parental strains and analysed for yeast uptake (A) maximal intracellular yeast cell proliferation (IPR) (B) and occurrence of cryptococcal expulsion (C). No statistically significant differences were found between the GFP-expressing strains and their parental counterpart (Table S1). Data are presented as mean (n≥3) with 2 times standard error (A+B) or accumulated data from three independent repeats (C).

Since macrophage/cryptococcal interactions are critical for disease progression [26], [27], we tested whether the expression of GFP altered cryptococcal behaviour within phagocytic effector cells. Using the J774 macrophage-like cell line, we tested each of the GFP positive strains for a) phagocytic uptake (Figure 3A), b) intracellular proliferation (Figure 3B) and c) the occurrence of expulsion (Figure 3C). All of the GFP-expressing strains were indistinguishable from their parental strains in each parameter tested (Table S1). Thus, expression of GFP in these strains does not alter their ability to parasitize macrophages.

### GFP expression is entirely distinct from *Cryptococcus* autofluorescence

**Figure 4:**
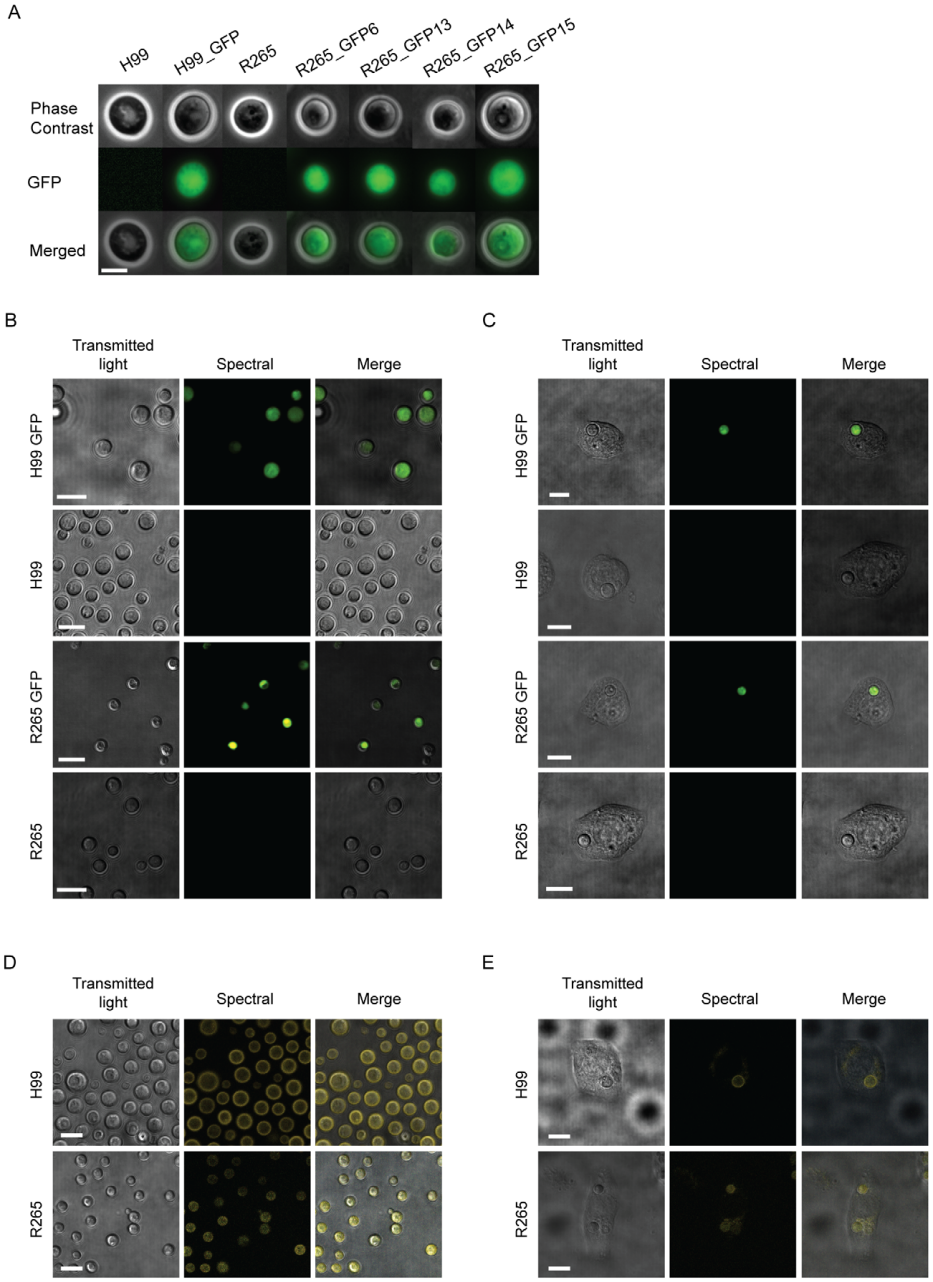
Imaging analysis of cryptococcal GFP fluorescence and autofluorescence. (A) GFP expressing strains exhibit a strong fluorescence signal that is absent in the parental strains. Epifluorescence images were captured and processed identically for each parental strain and transformant. (B and C) Spectral confocal images of parental strains and transformants showing absence of observable green fluorescence in parental strains when compared to the GFP expressing transformants either alone (B) or after phagocytosis by a macrophage (C). (D and E) Spectral confocal images of autofluorescence of parental strains either alone (D) or after phagocytosis by a macrophage (E). Spectral images were captured over 32 channels, 6 nm apart, between 500.1 nm and 691.3 nm. Spectral images images have been coloured to their actual fluorescent spectra. For separate channels of spectral images see Figures S2, Figures S3, Figures S4, Figures S5, Figures S6, Figures S7, Figures S8, Figures S9, Figures S10, Figures S11, Figures S12, Figures S13. Scale bars are 5 µm.

Imaging by epifluorescence showed that GFP expressing strains expressed strong cytoplasmic green fluorescence that was entirely absent from parental strains (Figure 4A). To distinguish between GFP fluorescence and autofluorescence we imaged our strains by spectral confocal microscopy. In agreement with our epifluorescence data, with identical image capture and processing, the parental strains had no discernable fluorescence relative to the GFP expressing strains (Figure 4B). After phagocytosis of yeast by macrophages there was no change in fluorescence in either the GFP expressing strains or the parental strains (Figure 4C). Furthermore, time lapse imaging of intracellular H99 for 9 hours resulted in no observable increase in autofluorescence in comparison to the fluorescence of the GFP expressing H99 strain (Figure S1). The autofluorescence of our two parental strains was only visible with a 5-fold increase in laser power and maximum detector sensitivity (Figure 4D). This autofluorescence was not visibly altered after phagocytosis by macrophages (Figure 4E). In addition, we include here the 32 individual, 6 nm wavelength channel images for each strain, both intracellular and extracellular, and note that the spectra of GFP and *Cryptococcus* autofluorescence are sufficiently different to allow spectral un-mixing when, unlike in our strains, GFP fluorescence is too weak to allow separation by intensity (Figures S2–12).

### GFP expressing strains can be used for automated analysis of cryptococcal interaction with macrophages

**Figure 5:**
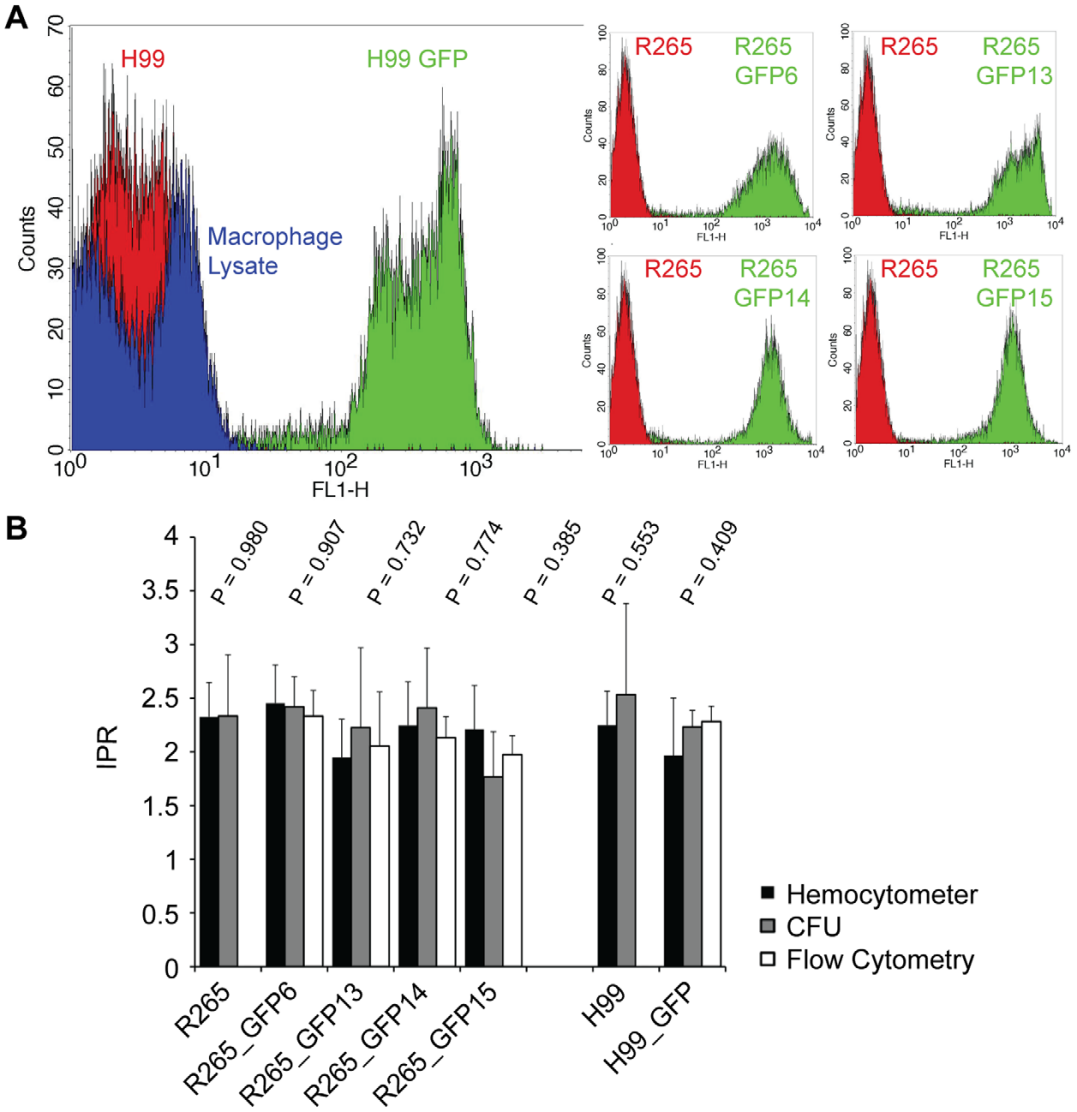
Automated counting of intracellular *Cryptococcus* proliferation within macrophages. (A) GFP-tagged strains were first analysed for their expression profile. All GFP-expressing strains are clearly discernable from GFP-negative cryptococci or cell debris. (B) Replicate samples were used for quantifying intracellular yeast cell numbers after macrophage infection via haemocytometer (black bars), CFU count (grey bars) or flow cytometry (white bars). For all of the strains, there was no statistically significant difference between any of the three counting methods. Data are presented as means with 2 times standard error from at least three independent repeats.

To date, analysis of cryptococcal interactions with macrophages has required elaborate and time-consuming experimental approaches. Potentially, flow cytometric-based systems offer an accurate but high-throughput alternative strategy for analysing intracellular proliferation. Since the GFP-positive strains described above are unaltered in their behaviour in macrophages, we used these strains to develop a flow cytometry-based methodology for quantifying intracellular proliferation of cryptococci after lysis of mammalian cells. Analysis of fluorescence and forward scatter by flow cytometry showed all five GFP expressing strains were clearly distinct from both the non-GFP expressing parental strains and from uninfected macrophages (Figure 5A). Similar data were obtained for R265-GFP (data not shown). Our previously published method for assaying proliferation within macrophages requires lysis of macrophages followed by manual counting of numerous replicate samples using either a haemocytometer or by plating on YPD plates and counting colony forming units (CFU) [15], [28]. To validate our flow cytometry based method, we took replicate samples from macrophage infections with the same GFP-expressing strain and compared proliferation quantification by flow cytometry, CFU and haemocytometer count. Comparison of *Cryptococcus* proliferation from all three counting methods showed no significant differences. Thus, flow cytometry offers a reliable and rapid alternative to manual count-based methods of analysis (Figure 5B).

### A non-lytic method of quantifying macrophage-*Cryptococcus* interaction by flow cytometry

**Figure 6:**
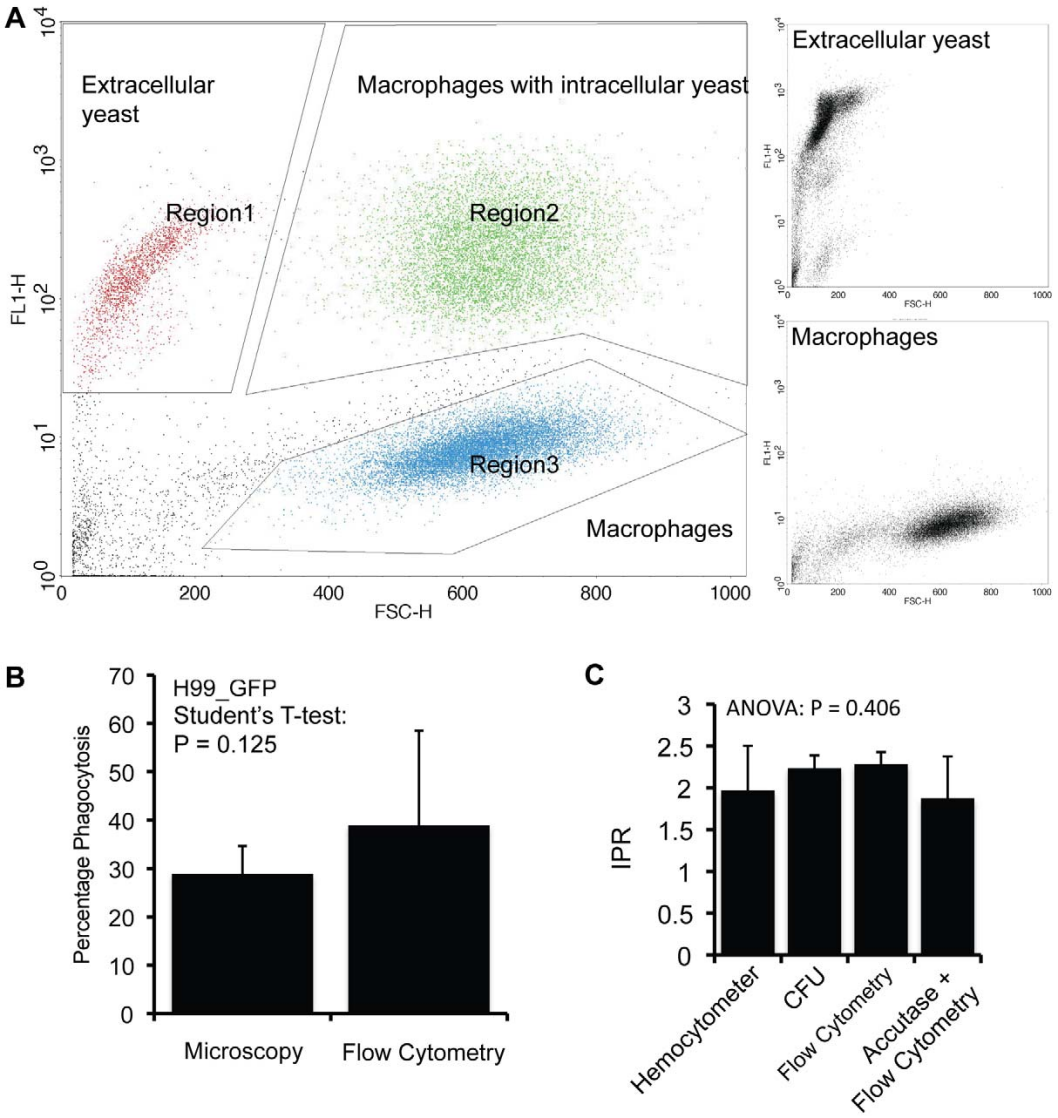
Assessment of virulence parameters in the macrophage model system after treatment with Accutase. Macrophages were infected with the GFP-positive H99 strain H99_GFP and cultured in the same way as for a normal infection assay. Samples were taken at standard time points but instead of cell lysis, the cultures were treated with Accutase to detach and dissociate cells. (A) Extracellular yeast, uninfected macrophages and macrophages with intracellular yeast cells show three distinct populations in a scatter plot. (B) Phagocytic uptake can be quantified by comparing the proportions of infected and uninfected macrophages using flow cytometry. Results are not statistically different between the two approaches. (C) Intracellular proliferation can also be determined with this approach by calculating fluorescence intensity. Results are not statistically different from IPR measurements with a haemocytometer, by CFU counts or flow cytometry counting after macrophage lysis. Data are presented as means with 2 times standard error from at least three independent repeats.

To date, all methods for quantifying intracellular cryptococcal proliferation involve a lysis step to free any intracellular yeast cells for counting. Lysis is methodologically undesirable since it increases processing time, increases the risk of sample loss and may potentially damage intracellular cryptococci. Accutase, a proteolytic and collagenolytic enzyme mix, allows detachment and dissociation of mammalian cells without significantly influencing viability from culture plates *in vitro* [29] potentially enabling us to quantify fungal burden within infected macrophages without lysis. J774 macrophage cultures were infected with H99_GFP, incubated according to the protocol for IPR assays and then treated with the enzyme mix instead of being lysed. Flow cytometry demonstrated that extracellular yeast cells, macrophages and macrophages with intracellular yeast cells formed three distinct regions based upon their size (forward scatter, FSC) and GFP fluorescence intensity (Figure 6A). Using fluorescence activated cell sorting we separated the region of macrophages containing intracellular yeast. Assessment of the purity of this region by microscopy revealed that this population contained >90% macrophages with intracellular yeast. By comparing events counted for uninfected macrophages versus events counted for macrophages with intracellular cryptococci we could accurately quantify percentage phagocytosis (Figure 6B). In addition, by calculating fluorescence intensity, we were able to quantify intracellular cryptococcal proliferation without the need to lyse the macrophage culture (Figure 6C).

## Discussion

Here we describe the generation of *Cryptococcus* type strains expressing GFP; *C. gattii* GFP-positive serotype B R265 strains and a *C. neoformans* GFP-positive serotype A H99 strain. Despite strong, stable GFP expression, these strains are indistinguishable from non-fluorescent parental isolates in terms of growth rate, stress tolerance or their ability to parasitize macrophages. We then used these strains to develop and validate a flow cytometry based assay for quantifying intracellular cryptococcal proliferation. The fluorescence of our GFP expressing cells far exceeds any autofluorescence, even when intracellular, and in addition can be spectrally distinguished by appropriate confocal techniques if, unlike in our strains, GFP fluorescence is at a similar level as autofluorescence. Endogenous GFP expression has substantial advantages over exogenous fluorescent labels since it does not require any secondary manipulations such as staining or treatment with a probe and thus can be directly used for analysis. Although GFP is generally regarded as a protein that does not influence cellular processes, it is known that in *Salmonella enterica* expression of GFP alters establishment of the intracellular niche in epithelial cells and macrophages [30]. However, careful analysis indicates that the GFP expressing cryptococci reported here show no significant differences in their stress responses or behaviour within macrophages.

GFP and its molecular cousins [31] offer many advantages over dye labelling previously used to track *Cryptococcus in vivo* and *in vitro* [19], [20], [32], [33], [34]. For example the commonly used dye FITC is quenched in acidic conditions, a major disadvantage for studies in macrophages. In addition, a number of these dyes show photoxicity in comparison to GFP, a major impediment to live imaging. Our GFP is stably expressed and is not diluted by proliferation, making long term study *in vivo* a possibility. Furthermore, multi-colour expression systems have been established that could be easily adapted to allow the tracking of many members of an infective population simultaneously [35].

The interaction with macrophages plays a key role in the outcome of cryptococcal infections. After phagocytosis, *Cryptococcus* manipulates host macrophages to establish itself as an intracellular parasite that can survive and proliferate within the macrophage phagolysosome [10]. This intracellular niche might help to explain how *Cryptococcus* stays latent within infected individuals and also how the yeast disseminates within the host. Phagocytosis, intracellular proliferation and the ability to exit macrophages in a non-lytic manner (expulsion) are important parameters in understanding *Cryptococcus*-macrophage interactions [9], [13], [14], [15], [17], [18], [28], [36], [37], [38], [39], [40], [41], [42], [43], [44], [45], [46], [47], [48], [49], [50], [51], [52], [53], [54], [55], [56]. In particular, the ability to proliferate within these host cells is an important pathogenesis feature as it correlates with *in vivo* virulence data from mice [15]. However, to date the analysis of intracellular proliferation and yeast uptake by phagocytes has required time consuming methods, thus preventing high throughput screening or related experimental approaches. The data presented here describe GFP-positive derivatives of widely used *Cryptococcus* strains coupled with a flow cytometry approach that enables efficient and high-throughput analysis of virulence parameters in the macrophage model system. Flow cytometry allows the analytical, and physical (by fluorescently activated cell sorting), separation of infected macrophages and therefore this population can be separately assessed for immune regulatory changes. Furthermore, our methodology potentially permits high throughput examination of drugs and anti-fungal components from both new and existing libraries, and, critically, the ability to analyse within the host macrophage environment.

In summary, we report a reliable tool for rapid and simple quantification of cryptococcal macrophage parasitism, which can easily be transferred to other *in vivo* and *in vitro* model systems.

## Materials and Methods

### Biolistic transformation

*C. neoformans* serotype A strain H99, originally isolated from patient cerebrospinal fluid, and *C. gattii* serotype B strain R265, a clinical isolate from an outbreak on Vancouver Island, Canada, were transformed with a GFP construct by biolistic bombardment [57]. The insertion cassette was constructed by PCR amplification with overlapping primers (Table 1) of the *C. neoformans* JEC21 *act1* promoter (AY483215), the GFP coding sequence and the *C. neoformans* JEC21 *trp1* terminator (AY483215) followed by lithium acetate transformation in the uracil negative *Saccharomyces cerevisiae* strain MLY40 [58] to achieve homologous recombination of overlapping DNA fragments into the plasmid pRS426. The plasmid was recovered from *S. cerevisiae*, electroporated into *Escherichia coli* DH5α and the GFP construct isolated by PCR (Table 1). After digestion with XhoI and BamHI the promoter/GFP/terminator cassette was ligated into the shuttle vector pAG32 [23] for biolistic DNA delivery. The GFP construct containing shuttle vector pAG32 was directly used for biolistic transformation. For biolistic bombardments, parental strains H99 and R265 were grown overnight in 50 ml YPD media at 30°C and 180 revolutions per minute in a shaker. Cells were collected by centrifugation for 5 minutes at 900 rcf and washed with dH_2_O. Yeast cells were resuspended in ¼ of the original volume, 300 µl aliquots spread in the centre of 1 M sorbitol YPD plates and left to dry at 30°C. Ten µl of gold particles (0.25 g of 0.6 µm beads in 750 µl ethanol) were mixed with 2 µl of 1 µg/µl DNA, 10 µl 2.5 M CaCl_2_ and 2 µl of spermidine-free base and incubated for 5 minutes at room temperature to attach the DNA to the gold particles. Free DNA was removed by a wash step with ethanol, the bead-DNA complexes resuspended in 12 µl ethanol and applied to the centre of a microcarrier biolistic disc (2.5 cm, pre-washed in ethanol, BioRad). The gold microcarrier were accelerated in a helium-generated vacuum (1350psi rupture discs, BioRad) and shot onto the 1 M sorbitol plates containing *Cryptococcus* in a BioRad PDS-1000/He biolistic particle delivery system. After transformation, yeast cells were recovered for 5 hours at 30°C, the cells lawn washed off with 1 ml liquid YPD media, plated on selective media containing 250 µg/ml hygromycin B and incubated for 5 days at 30°C. Three individual transformations were carried out with 10 plates being objected to biolistic bombardment each time [57].

**Table 1:** Primer.

Fragment	Sequence (5′-3′)
*Act1* promoter	Forward: TTGGGTACCGGGCCCCCCCTCGAGGTCGACGGTATCGATAAGGCTGCGGGAGGTGAGCTGG
	Reverse: TCCTCGCCCTTGCTCACCATAGACATGTTGGGCGAGTTTTAC
GFP coding sequence	Forward: GTAAAACTCGCCCAACATGTCTATGGTGAGCAAGGGCGAGGAG
	Reverse: CCTTACGGCCTTCACAATTACTTGTACAGCTCGTCCATGCCG
*Trp1* terminator	Forward: CGGCATGGACGAGCTGTACAAGTAATTGTGAAGGCCGTAAGG
	Reverse: AGAACTAGTGGATCCCCCGGGCTGCAGGAATTCGATATCAGAAGAGATGTAGAAACAGTTTCG
GFP construct	Forward: GTAGGATCCAGGCTGCGGGAGGTGAGCTGG
	Reverse: TAGGATCCGAAGAGATGTAGAAACGAGTTTCG

Primers used for construction of the GFP cassette and biolistic transformation in *Cryptococcus* strains.

### Yeast cells and growth conditions

Cryptococcal strains were incubated in liquid YPD media (1% peptone, 1% yeast extract, 2% D-(+)-glucose) for 24 hours at 25°C on a rotator at 20 revolutions per minute prior to experimental use. Fluorescence images of yeast cells were taken after growth in YPD media overnight at 25°C on a rotator at 20 revolutions per minute with a Zeiss Axiovert 135 TV microscope with 100 x oil immersion Plan-Neofluor objective.

### Stress treatment

GFP expressing strains were tested for their susceptibility towards cellular stresses with the following conditions: hypoxia (3%, CoCl_2_), oxidative (H_2_O_2_), nitrosative (NaNO_2_) and cell wall (sodium dodecyl sulphate (SDS) and NaCl) in Dulbecco's modified Eagle's medium (DMEM) supplemented with 2 mM L-glutamine, 100 U/ml penicillin and 100 U/ml streptomycin (assay medium), the medium routinely used in macrophage assays. Cryptococcal cells from 24 hours cultures were washed three times with phosphate buffered saline (PBS), counted in a haemocytometer and adjusted to 10^5^ cells per ml in assay medium. One ml of yeast solution was incubated with the appropriate stress in 48-well plates and left at 37°C and 5% CO_2_ for 24 hours without shaking. To assess the influence of stress conditions on cryptococcal growth, serial dilutions were plated and colony-forming units (CFUs) counted after 0 and 24 hours. CFUs relative to time point 0 were calculated.

### Mammalian cells and growth conditions

The macrophage-like cell line J774 [59] was used for this study and growth conditions were as described before [15], [28]. The cells were used between passage 5 and 20 after thawing and cultured in Dulbecco's modified Eagle's medium (DMEM) supplemented with 2 mM L-glutamine, 100 U/ml penicillin and 100 U/ml streptomycin and 10% fetal bovine serum (culture medium) at 37°C and 5% CO_2_.

### Macrophage virulence assays

Infections of macrophages with *Cryptococcus*, phagocytosis assays, proliferation assays and live-cell imaging was performed as described before [15], [28]. Briefly, 1 ml of J774 cells (10^5^ cells/ml) in culture medium was plated into 24 well plates and incubated for 24 hours at 37°C and 5% CO_2_ before start of the assay. Cells were activated with 1 mg/ml of the immune stimulant phorbol myristic acetate (PMA) in assay medium (as culture medium but without 10% fetal bovine serum) for 1 hour at 37°C and 5% CO_2_. At the same time, yeast cells from 24 hours cultures were washed three times with PBS and opsonized with 10 µg/ml of 18B7 antibody (a gift of Arturo Casadevall) for 1 hour at 37°C. Then, macrophage infection with yeast cells (1∶10 ratio) was allowed to proceed for 2 hours in assay medium at 37°C and 5% CO_2_. After this incubation time, uninternalized yeast cells were removed by extensive washing with PBS and the effectiveness of washing assessed by light mircrosopy. For assessment of phagocytosis, macrophages were plated on acid-washed (1 M HCl) 13 mm glass coverslips and, after infection, fixed with 4% paraformaldehyde for 20 minutes at 4°C. The coverslips were washed with PBS and dH_2_O and then mounted with Mowiol (Calbiochem, Nottingham, UK) mounting media (100 mM Tris-HCl, pH 8.5, 9% Mowiol, 25% glycerol) onto glass slides. At least 1,000 cells were assessed for intracellular yeast cells and percentage phagocytosis calculated as percentage of cells with internalized *Cryptococcus*. For intracellular proliferation, infected macrophages were further incubated in assay medium at 37°C and 5% CO_2_ and samples taken after 0, 18, 24, 48 and 72 hours. Extracellular yeast were removed by washing with 200 µl PBS and collected, intracellular yeast cells were collected by macrophage lysis in a total of 200 µl dH_2_O and number of yeast cells counted with a haemocytometer, by plating serial dilutions and assessing CFU and by flow cytometry. Yeast cell numbers were compared to time point 0 and maximal intracellular proliferation (IPR) (typically after 24 hours) was used as measure for intracellular proliferative capacity (highest intracellular yeast cell number divided by the initial yeast count at time point 0). For live-cell imaging, infected macrophages were kept in assay medium and plates transferred into a control culture chamber (OKOLAB) at 37°C and 5% CO_2_. Images were taken every 90 seconds for 20 hours with a Nikon Digital Sight DS-Qi1MC camera on a Nikon Eclipse TE2000-U microscope with 20 x phase contrast objective and 1 x optivar and compiled to time-lapse movies using the software NIS-Elements AR 3.0. The number of expulsion events was counted by eye.

### Spectral confocal microscopy

Both yeast alone and macrophages with intracellular yeast were imaged live in 96-well imaging plates (BD Biosciences). Confocal images were captured on an A1-R instrument (Nikon) with 60 x objective (CFI Plan Apo TIRF oil 1.49NA) in galvo scanner mode with spectral detector in manual mode using NIS elements AR software (Nikon). The A1-R Z-axis was driven with a piezo drive (Mad City labs, Madison, WI). Spectral images were coloured and merged in NIS elements AR software (Nikon). Contrast was adjusted in Photoshop CS3 (Adobe). Laser and detector settings for each strain are given in the legend for Figures S2–13.

### Timelapse imaging

After phagocytosis of cryptococci, J774 macrophage cells were washed three times and imaged in DMEM without phenol red. Cells were imaged on a TE2000 (Nikon) microscope enclosed in a temperature controlled and humidified environmental chamber (OKOLAB) with 5% CO_2_ at 37°C. Time lapse images were captured with a Digital Sight DS-Qi1MC camera (Nikon), 20 x objective (Ph1 PLAN APO), using NIS elements AR software (Nikon). Images were captured every 2 minutes for 9 hours.

### Accutase treatment

J774 macrophages were detached and separated by Accutase (PAA) treatment. The cells were incubated with the undiluted proteolytic and collagenolytic enzyme mix for 15 minutes at 37°C and then gently dissociated by pipetting to ensure a single cell suspension; cell separation was checked by eye by light microscopy.

### Flow cytometry

Samples were fixed by adding an equal volume of 2% formaldehyde and 2% fetal bovine serum in PBS. Flow cytometry parameters were measured using a FACSCaliber instrument (BD Biosciences) and analysed with CellQuestPro (BD Biosciences). To enable comparison of different samples/time points, we assessed the number of events over a fixed time period. Intracellular proliferation was calculated by measuring the geometric mean of GFP fluorescence of macrophages containing cryptococci and this value normalised to the geometric mean of extracellular cryptococci (to adjust for any inherent variation in GFP florescence within the *Cryptococcus* population) and multiplied by the number of macrophages containing cryptococci. The resulting value for each time point was divided by the initial value at time point zero and the maximum ratio taken as the maximum proliferation rate.

### Fluorescently activated cell sorting of macrophages containing cryptococci

Five confluent 75 cm^2^ flasks of J774 macrophages were incubated with opsonised cryptococci for 2 hours, washed with PBS and detached using Accutase as described above. Cells were pelleted and resuspended in 3 mls of cell culture media. Cells were sorted, using a FACSAria II (BD Biosciences), into cell culture media and plated at 1×10^5^/ml.

### Statistical analysis

At least three individual repeats were performed for each experiment. Results for yeast growth, yeast stress response, IPRs and percentage phagocytosis were analysed for statistically significant differences using a one-way analysis of variance along with multicomparisons (Tukey's honestly significant difference test). For assessment of expulsion, results from at least three individual assays were tested for statistical significant differences using a Chi^2^- test. P-values of <0.05 were considered to be statistically significant.

## Supporting Information

Figure S1 Time lapse phase contrast and fluorescent images of intracellular H99 and H99_GFP were captured every 2 minutes for 9 ;hours. There is no observable change in H99 autofluorescence in comparison to the H99_GFP strain.
(TIF)

Figure S2 Individual spectral channel images of the H99 GFP strain. A1R confocal settings used were: 512×512 pixel scan area, 32 channels with 6 ;nm resolution between 500.1 ;nm and 691.3 ;nm, 1.2 ;mW 488 laser line, 166 spectral detector gain, 163 transmitted light detector gain, 0.08 ;µm/pixel.
(TIF)

Figure S3 Individual spectral channel images of the H99 strain. A1R confocal settings used were: 512×512 pixel scan area, 32 channels with 6 ;nm resolution between 500.1 ;nm and 691.3 ;nm, 1.2 ;mW 488 laser line, 166 spectral detector gain, 143 transmitted light detector gain, 0.09 ;µm/pixel.
(TIF)

Figure S4 Individual spectral channel images of the R265_GFP6 strain. A1R confocal settings used were: 512×512 pixel scan area, 32 channels with 6 ;nm resolution between 500.1 ;nm and 691.3 ;nm, 1.2 ;mW 488 laser line, 137 spectral detector gain, 167 transmitted light detector gain, 0.09 ;µm/pixel.
(TIF)

Figure S5 Individual spectral channel images of the R265 strain. A1R confocal settings used were: 512×512 pixel scan area, 32 channels with 6 ;nm resolution between 500.1 ;nm and 691.3 ;nm, 1.2 ;mW 488 laser line, 137 spectral detector gain, 143 transmitted light detector gain, 0.08 ;µm/pixel.
(TIF)

Figure S6 Individual spectral channel images of intracellular H99_GFP strain. A1R confocal settings used were: 512×512 pixel scan area, 32 channels with 6 ;nm resolution between 500.1 ;nm and 691.3 ;nm, 1.2 ;mW 488 laser line, 173 spectral detector gain, 140 transmitted light detector gain, 0.12 ;µm/pixel.
(TIF)

Figure S7 Individual spectral channel images of intracellular H99 strain. A1R confocal settings used were: 512×512 pixel scan area, 32 channels with 6 ;nm resolution between 500.1 ;nm and 691.3 ;nm, 1.2 ;mW 488 laser line, 173 spectral detector gain, 140 transmitted light detector gain, 0.10 ;µm/pixel.
(TIF)

Figure S8 Individual spectral channel images of intracellular R265_GFP strain. A1R confocal settings used were: 512×512 pixel scan area, 32 channels with 6 ;nm resolution between 500.1 ;nm and 691.3 ;nm, 1.2 ;mW 488 laser line, 145 spectral detector gain, 140 transmitted light detector gain, 0.10 ;µm/pixel.
(TIF)

Figure S9 Individual spectral channel images of intracellular R265 strain. A1R confocal settings used were: 512×512 pixel scan area, 32 channels with 6 ;nm resolution between 500.1 ;nm and 691.3 ;nm, 1.2 ;mW 488 laser line, 137 spectral detector gain, 167 transmitted light detector gain, 0.09 ;µm/pixel.
(TIF)

Figure S10 Individual spectral channel images of autofluorescence of H99 strain. A1R confocal settings used were: 512×512 pixel scan area, 32 channels with 6 ;nm resolution between 500.1 ;nm and 691.3 ;nm, 6 ;mW 488 laser line, 255 spectral detector gain, 110 transmitted light detector gain, 0.11 ;µm/pixel.
(TIF)

Figure S11 Individual spectral channel images of autofluorescence of R265 strain. A1R confocal settings used were: 512×512 pixel scan area, 32 channels with 6 ;nm resolution between 500.1 ;nm and 691.3 ;nm, 6 ;mW 488 laser line, 255 spectral detector gain, 110 transmitted light detector gain, 0.11 ;µm/pixel.
(TIF)

Figure S12 Individual spectral channel images of autofluorescence of intracellular H99 strain. A1R confocal settings used were: 512×512 pixel scan area, 32 channels with 6 ;nm resolution between 500.1 ;nm and 691.3 ;nm, 6 ;mW 488 laser line, 255 spectral detector gain, 110 transmitted light detector gain, 0.11 ;µm/pixel.
(TIF)

Figure S13 Individual spectral channel images of autofluorescence of intracellular R265 strain. A1R confocal settings used were: 512×512 pixel scan area, 32 channels with 6 ;nm resolution between 500.1 ;nm and 691.3 ;nm, 6 ;mW 488 laser line, 255 spectral detector gain, 110 transmitted light detector gain, 0.11 ;µm/pixel.
(TIF)

Table S1 Statistical Analysis. P‐values of statistical analysis of results from CFU counts from stress treatments and intracellular virulence assays of GFP‐positive strains compared to parental strains.
(DOC)
