# Supplementary figures and images for: Automated Analysis of Cryptococcal Macrophage Parasitism Using GFP-Tagged Cryptococci
Source: PLoS One. 2010 Dec 31;5(12):e15968. doi: 10.1371/journal.pone.0015968 (PMC3013146; doi:10.1371/journal.pone.0015968)

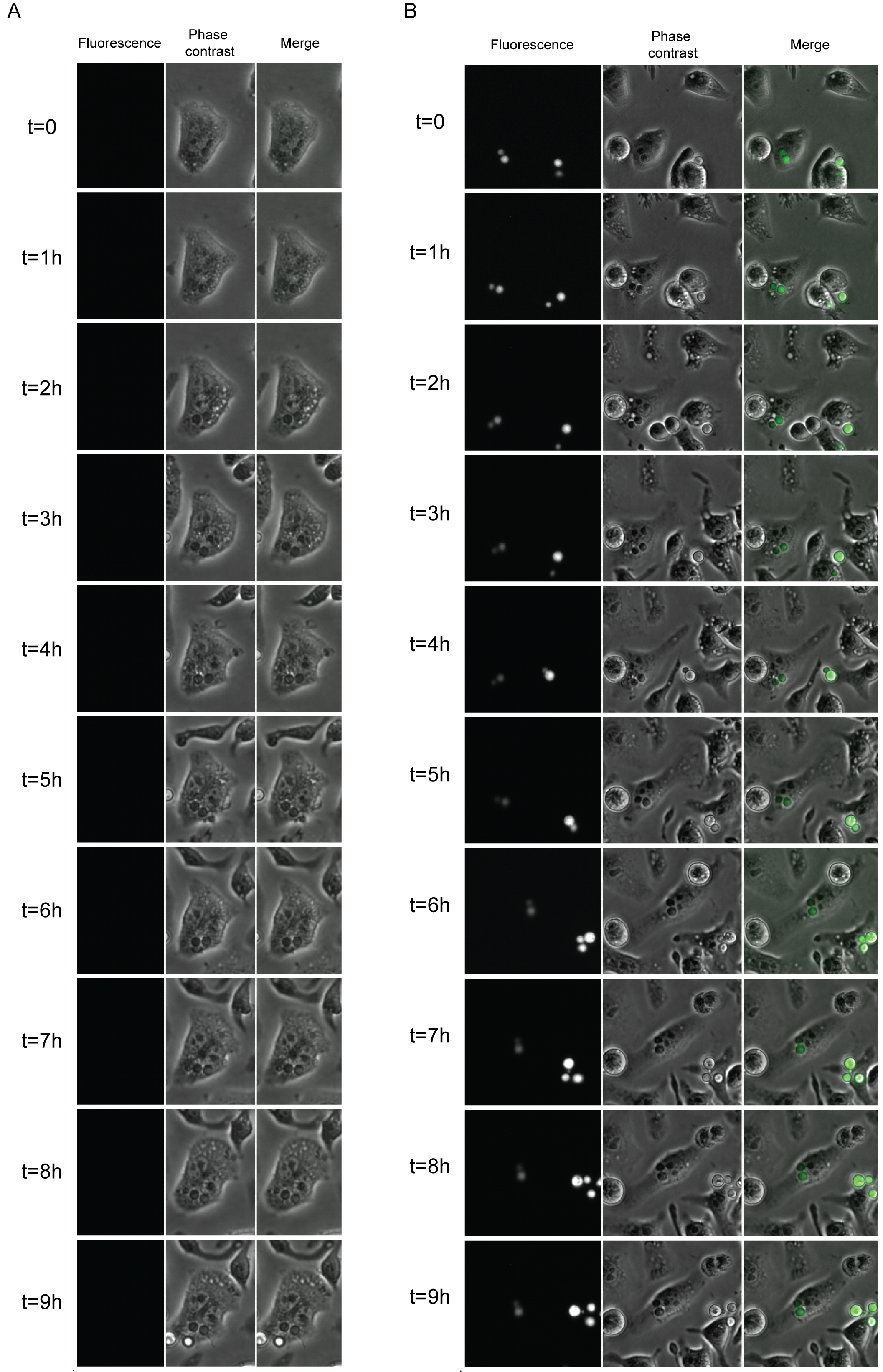

Supplement: Figure S1 — Time lapse phase contrast and fluorescent images of intracellular H99 and H99_GFP were captured every 2 minutes for 9 ;hours. There is no observable change in H99 autofluorescence in comparison to the H99_GFP strain. (TIF) [file pone.0015968.s001.tif]

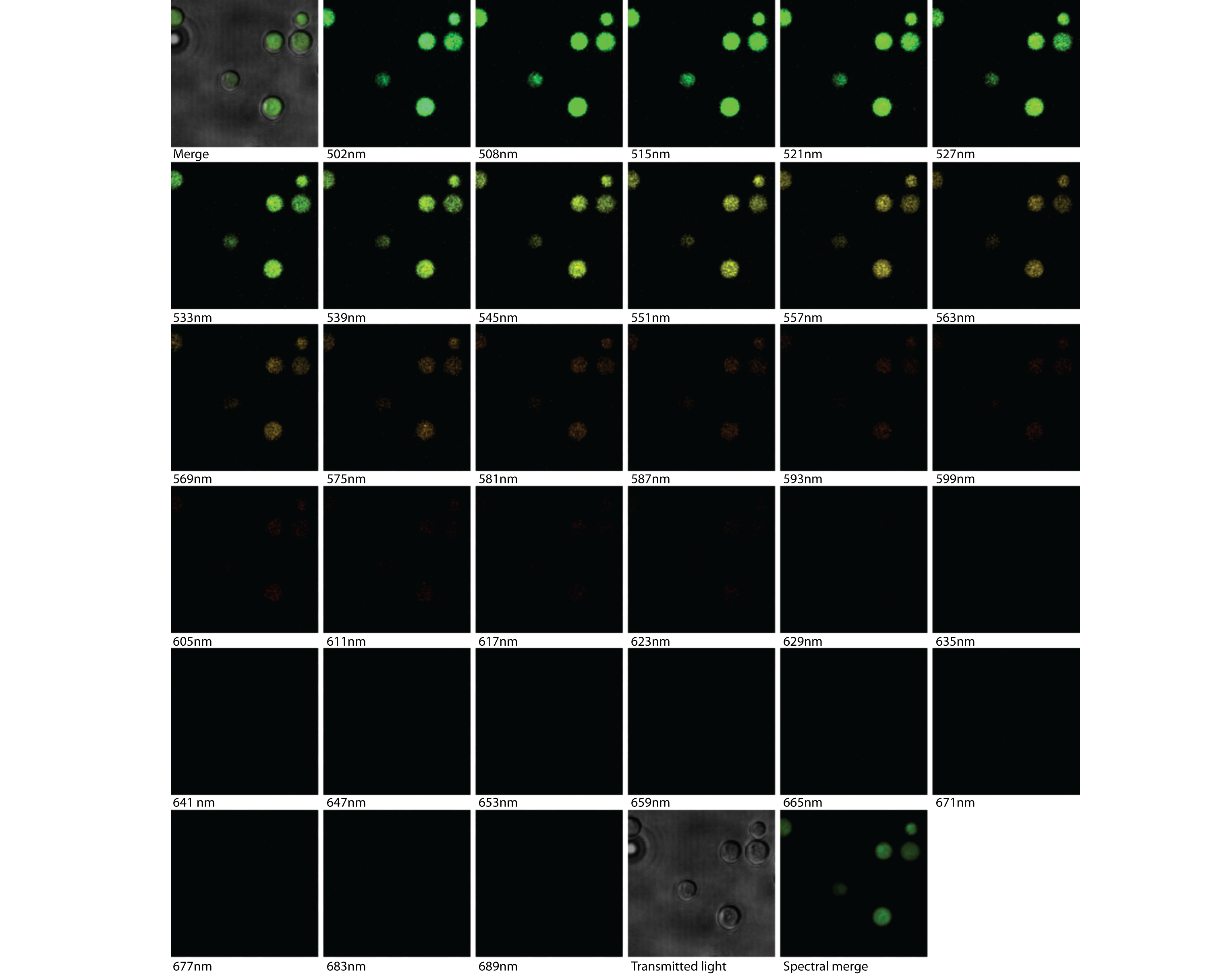

Supplement: Figure S2 — Individual spectral channel images of the H99 GFP strain. A1R confocal settings used were: 512×512 pixel scan area, 32 channels with 6 ;nm resolution between 500.1 ;nm and 691.3 ;nm, 1.2 ;mW 488 laser line, 166 spectral detector gain, 163 transmitted light detector gain, 0.08 ;µm/pixel. (TIF) [file pone.0015968.s002.tif]

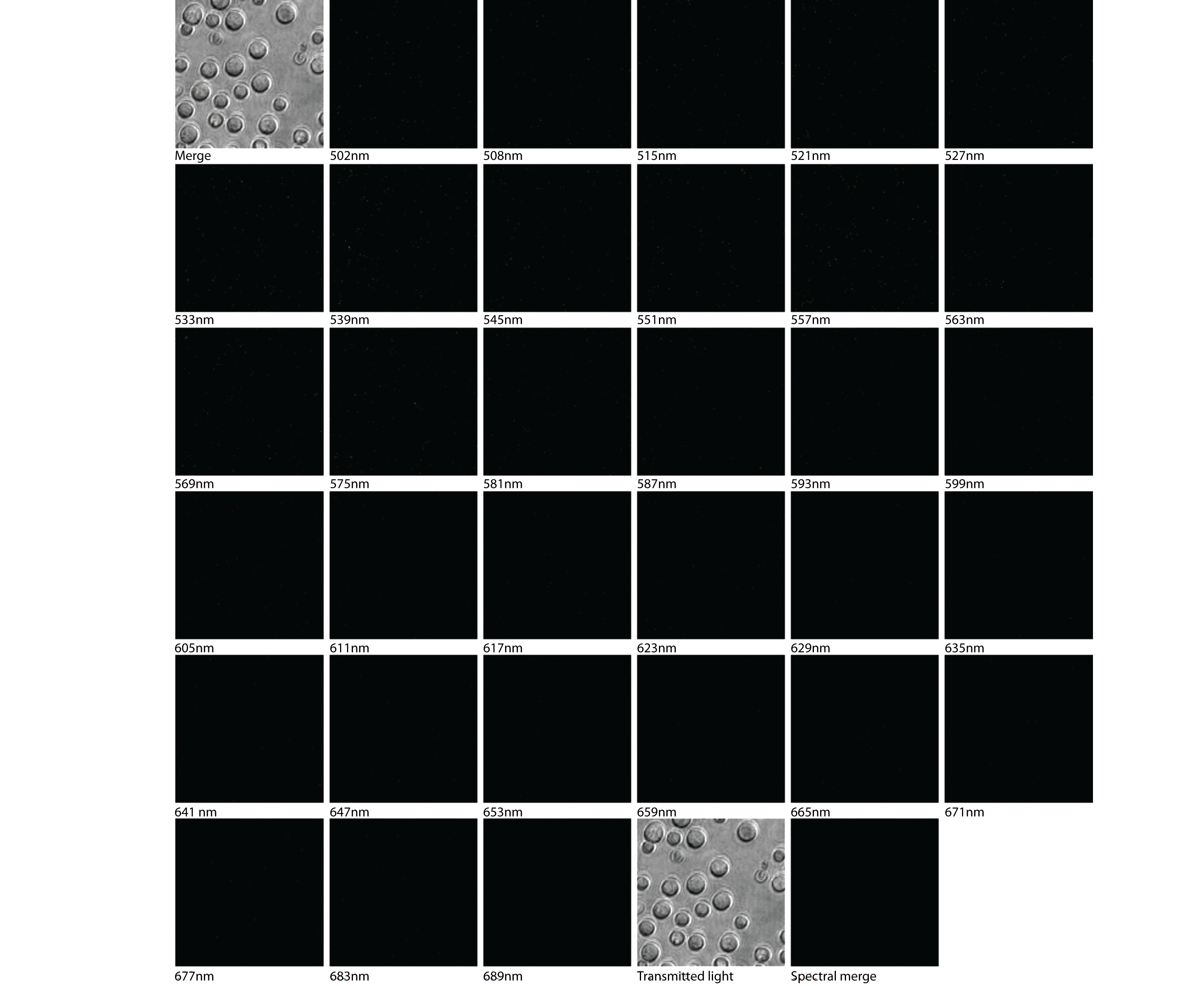

Supplement: Figure S3 — Individual spectral channel images of the H99 strain. A1R confocal settings used were: 512×512 pixel scan area, 32 channels with 6 ;nm resolution between 500.1 ;nm and 691.3 ;nm, 1.2 ;mW 488 laser line, 166 spectral detector gain, 143 transmitted light detector gain, 0.09 ;µm/pixel. (TIF) [file pone.0015968.s003.tif]

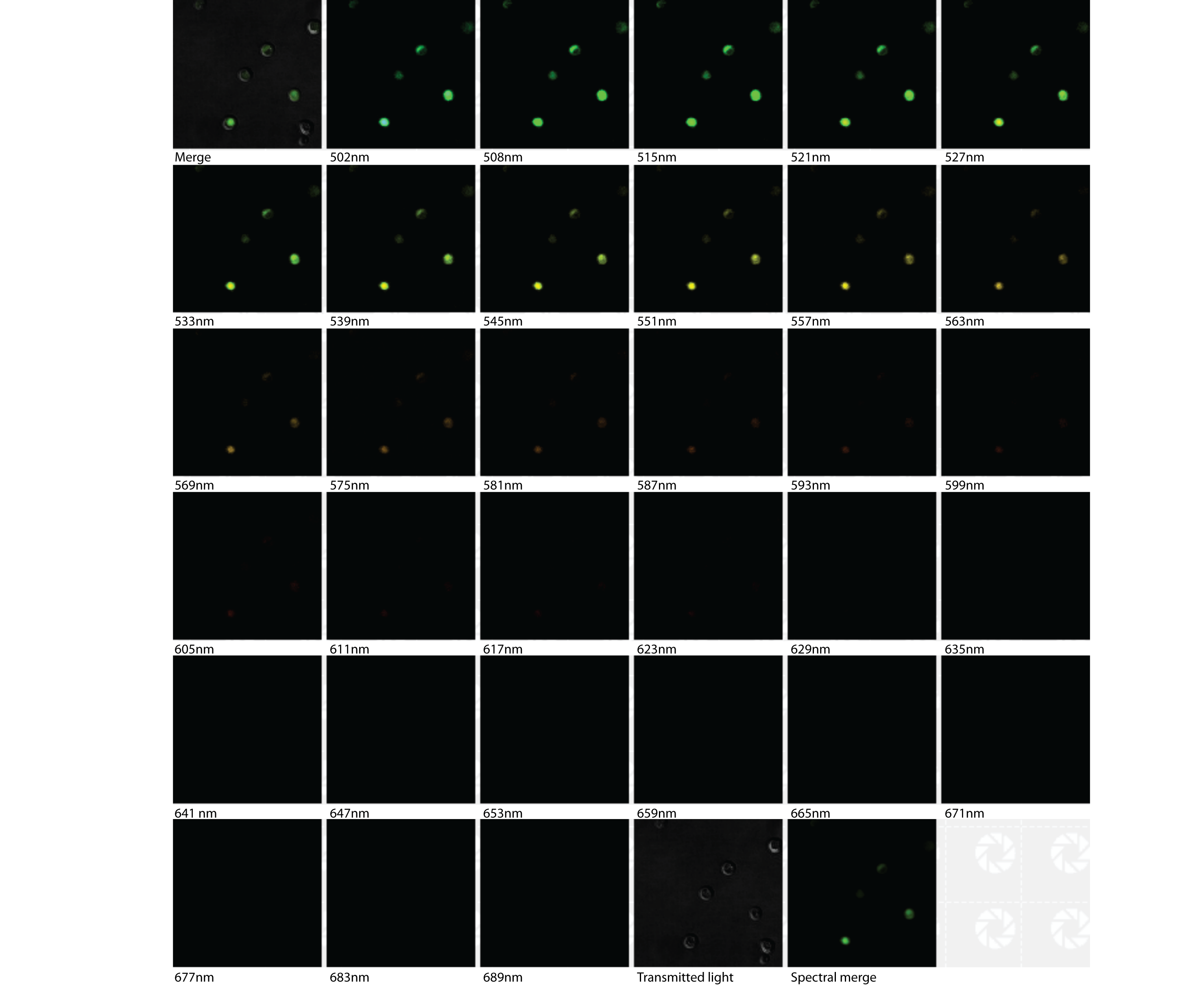

Supplement: Figure S4 — Individual spectral channel images of the R265_GFP6 strain. A1R confocal settings used were: 512×512 pixel scan area, 32 channels with 6 ;nm resolution between 500.1 ;nm and 691.3 ;nm, 1.2 ;mW 488 laser line, 137 spectral detector gain, 167 transmitted light detector gain, 0.09 ;µm/pixel. (TIF) [file pone.0015968.s004.tif]

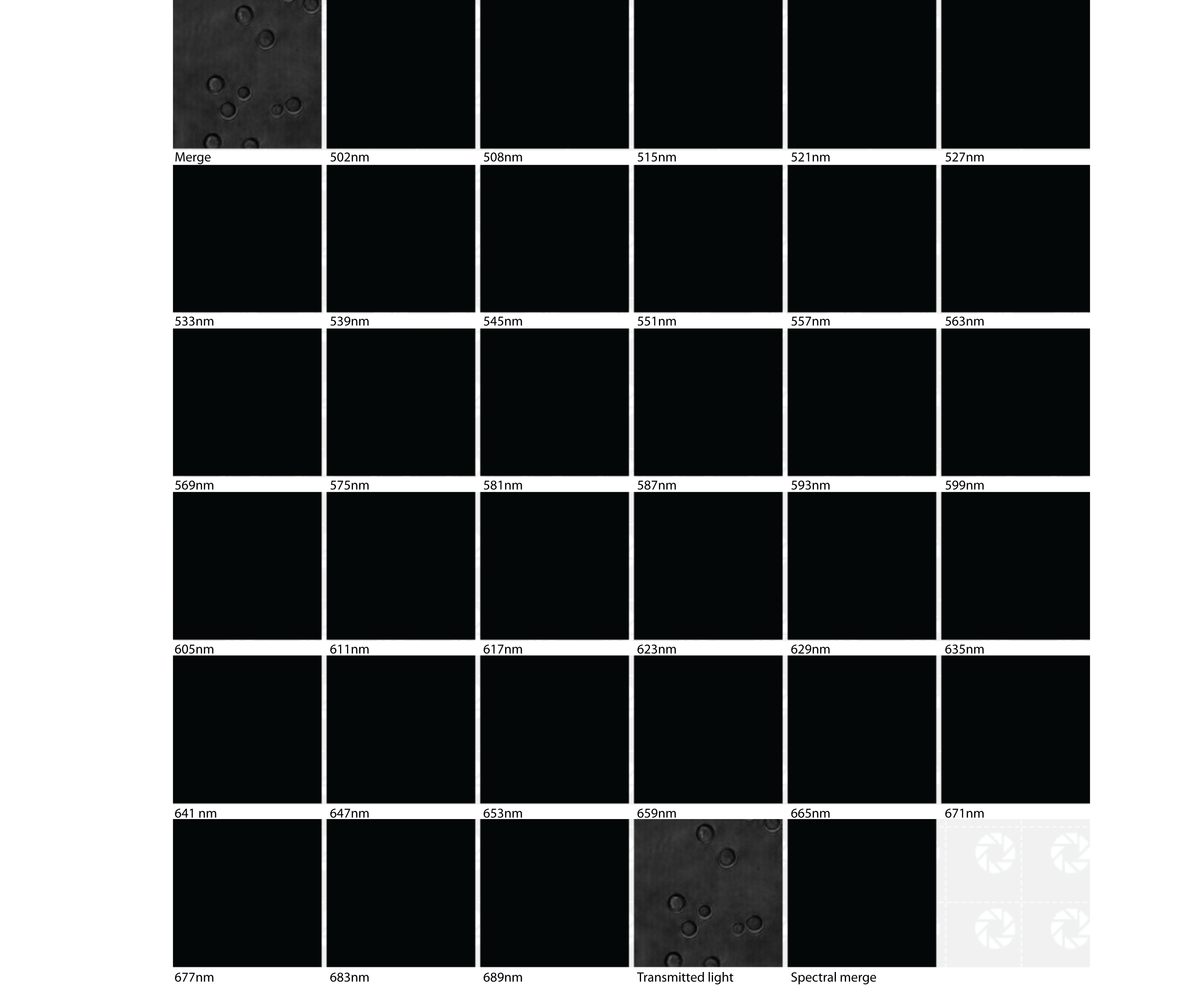

Supplement: Figure S5 — Individual spectral channel images of the R265 strain. A1R confocal settings used were: 512×512 pixel scan area, 32 channels with 6 ;nm resolution between 500.1 ;nm and 691.3 ;nm, 1.2 ;mW 488 laser line, 137 spectral detector gain, 143 transmitted light detector gain, 0.08 ;µm/pixel. (TIF) [file pone.0015968.s005.tif]

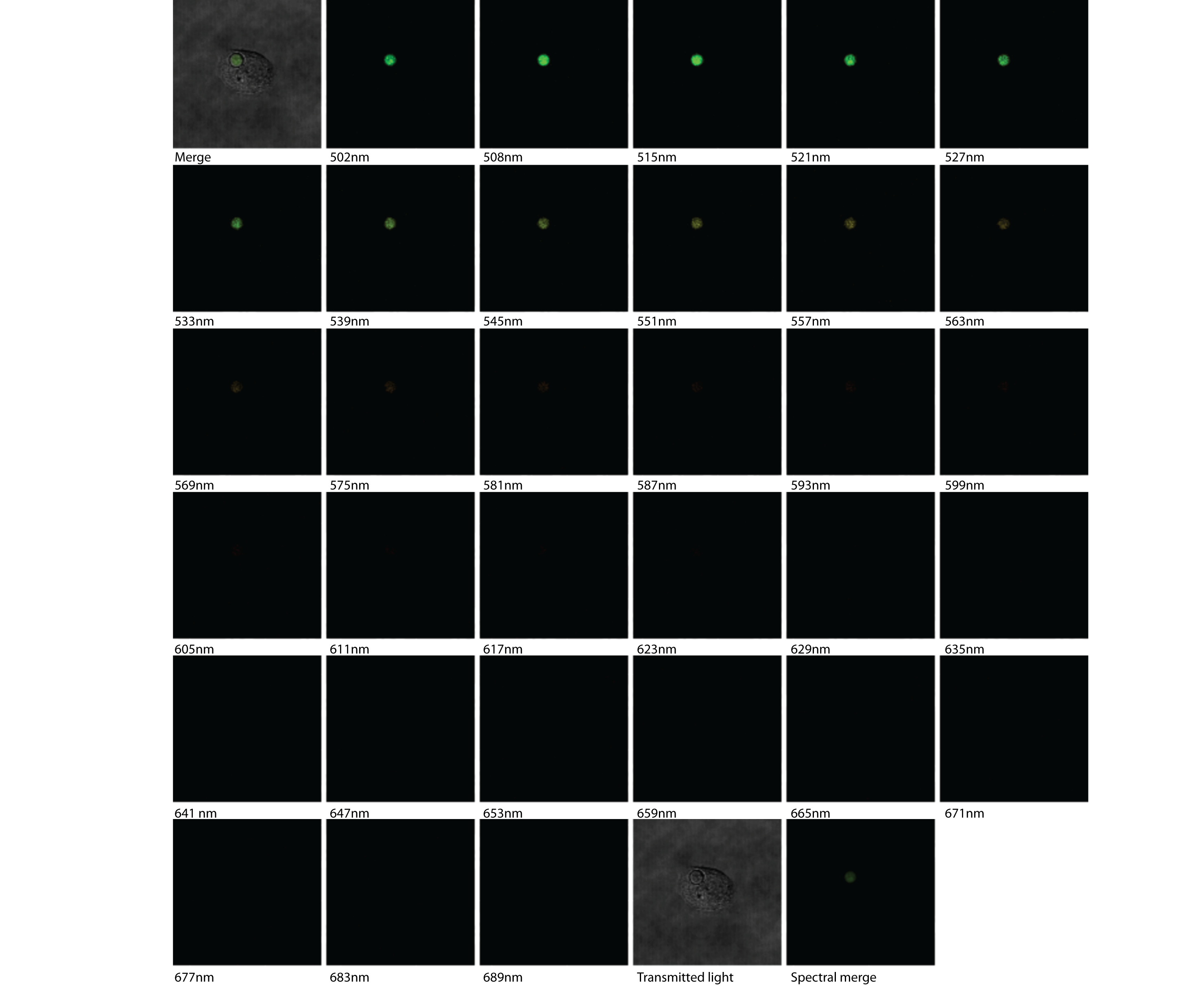

Supplement: Figure S6 — Individual spectral channel images of intracellular H99_GFP strain. A1R confocal settings used were: 512×512 pixel scan area, 32 channels with 6 ;nm resolution between 500.1 ;nm and 691.3 ;nm, 1.2 ;mW 488 laser line, 173 spectral detector gain, 140 transmitted light detector gain, 0.12 ;µm/pixel. (TIF) [file pone.0015968.s006.tif]

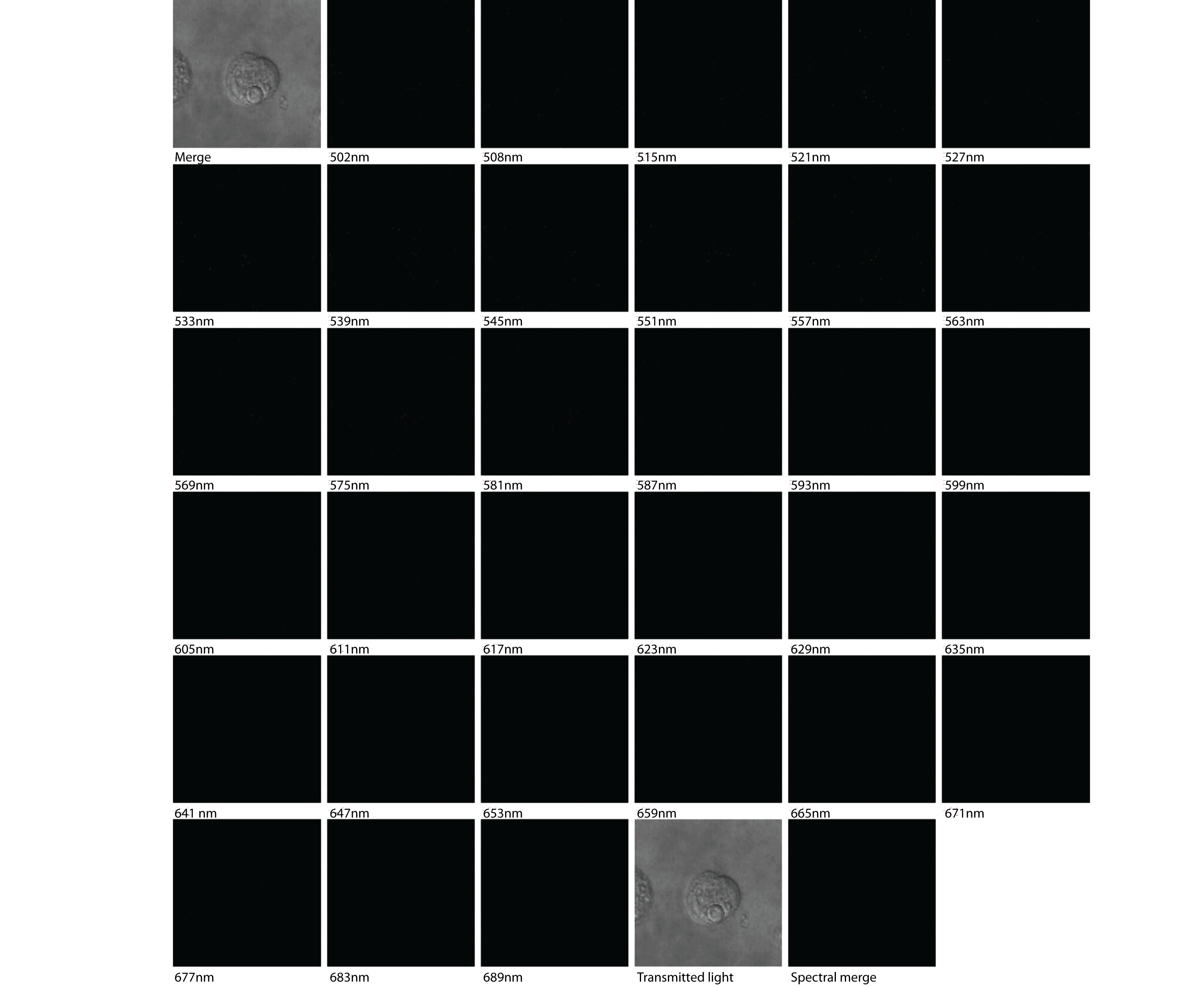

Supplement: Figure S7 — Individual spectral channel images of intracellular H99 strain. A1R confocal settings used were: 512×512 pixel scan area, 32 channels with 6 ;nm resolution between 500.1 ;nm and 691.3 ;nm, 1.2 ;mW 488 laser line, 173 spectral detector gain, 140 transmitted light detector gain, 0.10 ;µm/pixel. (TIF) [file pone.0015968.s007.tif]

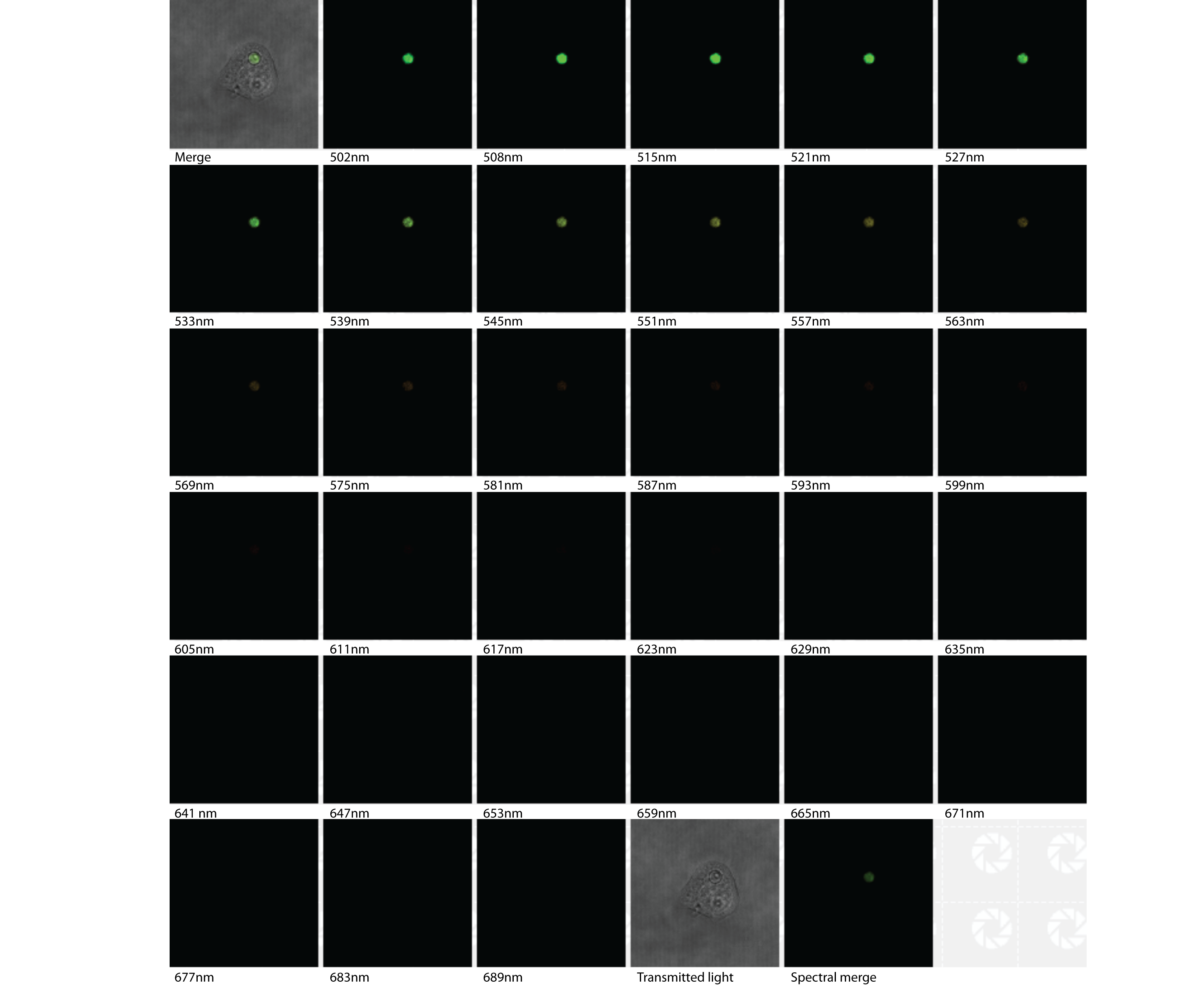

Supplement: Figure S8 — Individual spectral channel images of intracellular R265_GFP strain. A1R confocal settings used were: 512×512 pixel scan area, 32 channels with 6 ;nm resolution between 500.1 ;nm and 691.3 ;nm, 1.2 ;mW 488 laser line, 145 spectral detector gain, 140 transmitted light detector gain, 0.10 ;µm/pixel. (TIF) [file pone.0015968.s008.tif]

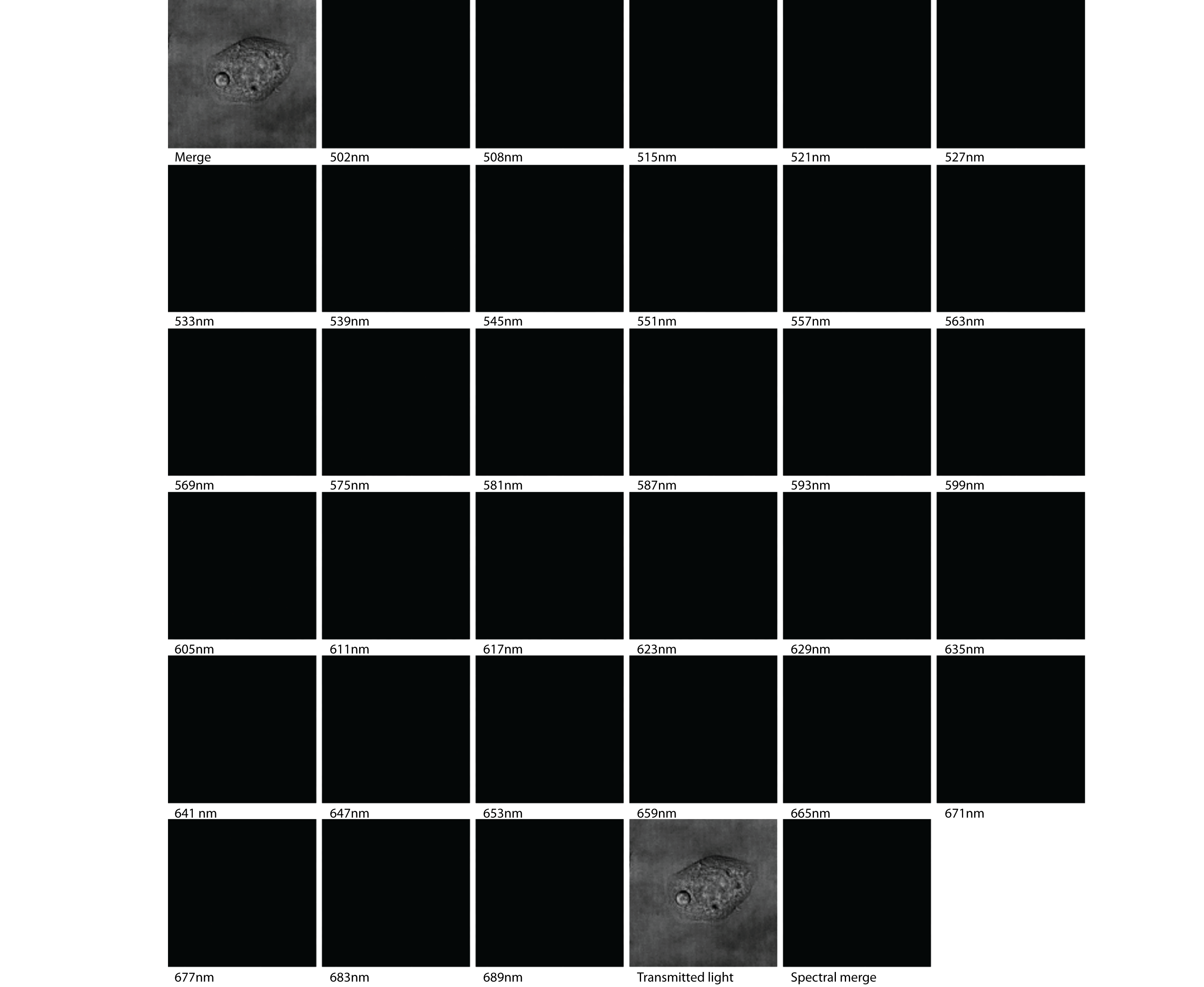

Supplement: Figure S9 — Individual spectral channel images of intracellular R265 strain. A1R confocal settings used were: 512×512 pixel scan area, 32 channels with 6 ;nm resolution between 500.1 ;nm and 691.3 ;nm, 1.2 ;mW 488 laser line, 137 spectral detector gain, 167 transmitted light detector gain, 0.09 ;µm/pixel. (TIF) [file pone.0015968.s009.tif]

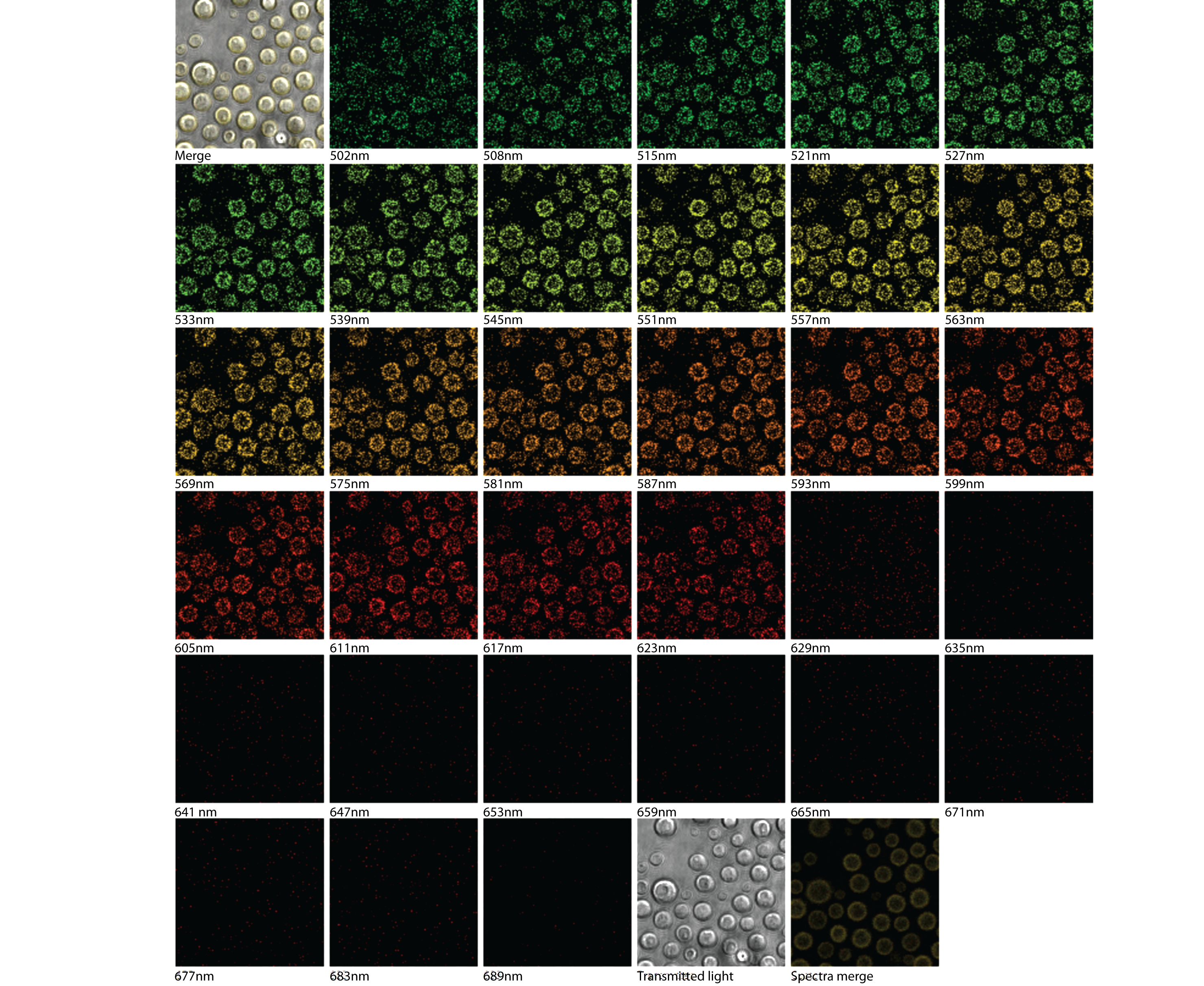

Supplement: Figure S10 — Individual spectral channel images of autofluorescence of H99 strain. A1R confocal settings used were: 512×512 pixel scan area, 32 channels with 6 ;nm resolution between 500.1 ;nm and 691.3 ;nm, 6 ;mW 488 laser line, 255 spectral detector gain, 110 transmitted light detector gain, 0.11 ;µm/pixel. (TIF) [file pone.0015968.s010.tif]

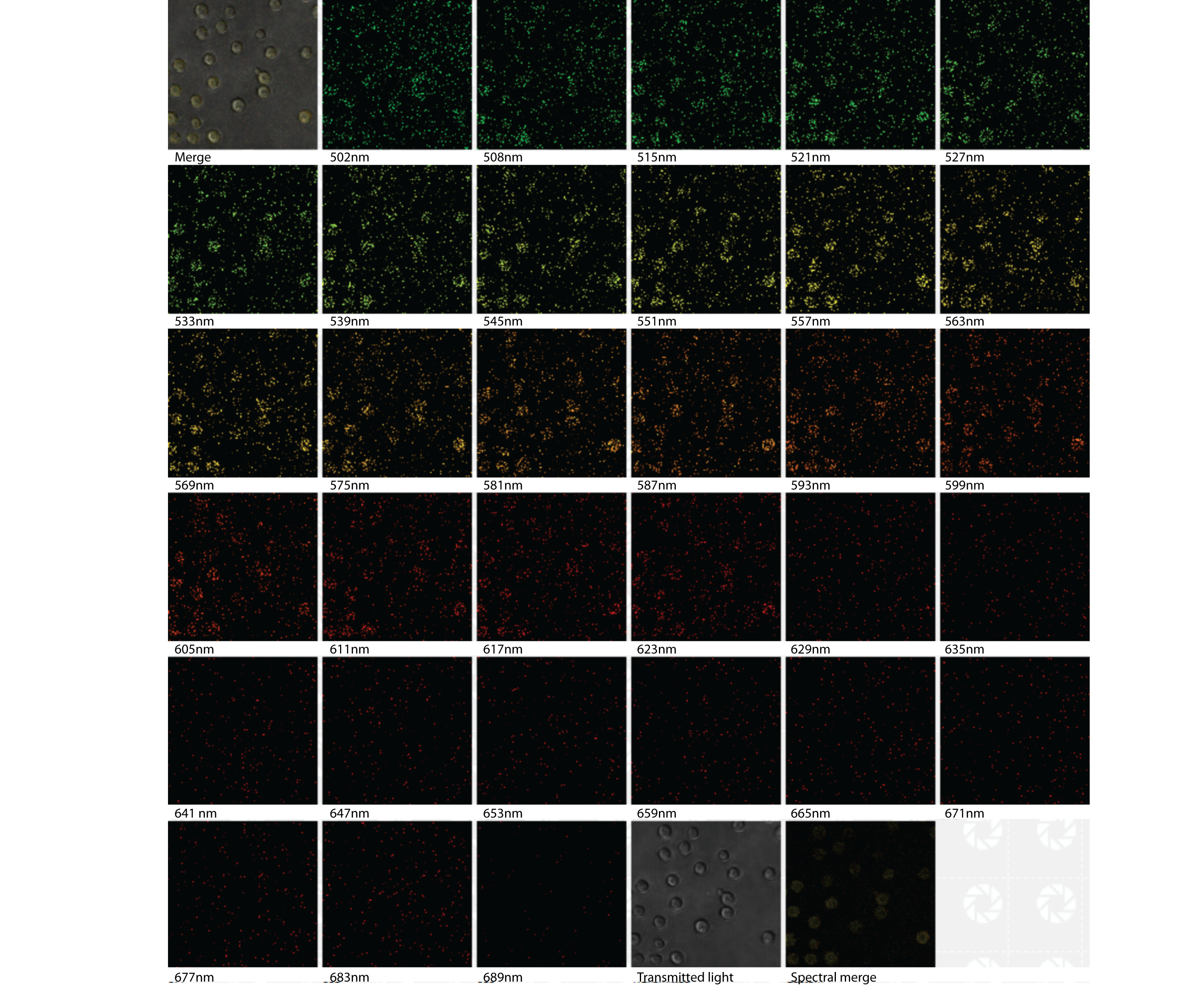

Supplement: Figure S11 — Individual spectral channel images of autofluorescence of R265 strain. A1R confocal settings used were: 512×512 pixel scan area, 32 channels with 6 ;nm resolution between 500.1 ;nm and 691.3 ;nm, 6 ;mW 488 laser line, 255 spectral detector gain, 110 transmitted light detector gain, 0.11 ;µm/pixel. (TIF) [file pone.0015968.s011.tif]

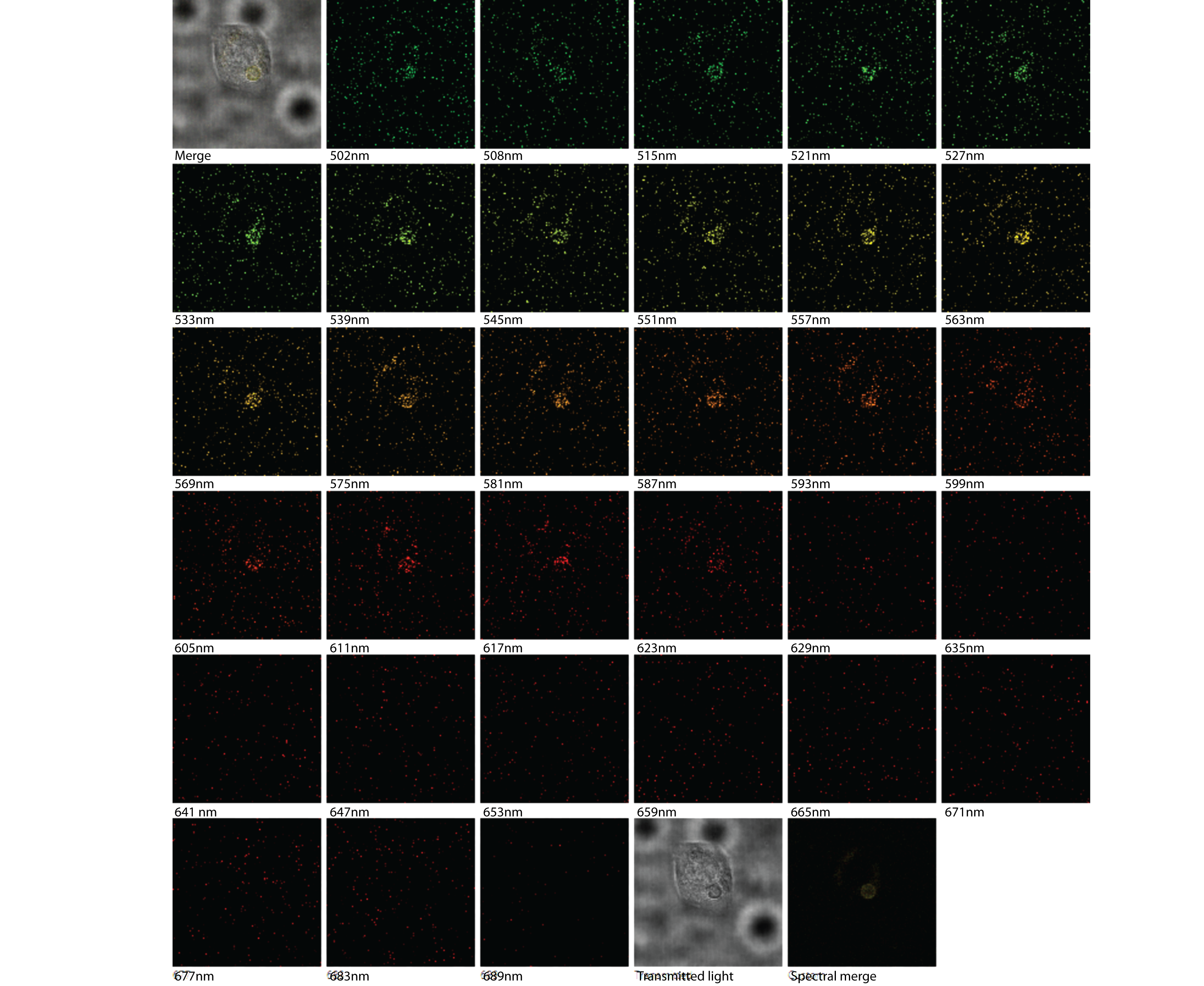

Supplement: Figure S12 — Individual spectral channel images of autofluorescence of intracellular H99 strain. A1R confocal settings used were: 512×512 pixel scan area, 32 channels with 6 ;nm resolution between 500.1 ;nm and 691.3 ;nm, 6 ;mW 488 laser line, 255 spectral detector gain, 110 transmitted light detector gain, 0.11 ;µm/pixel. (TIF) [file pone.0015968.s012.tif]

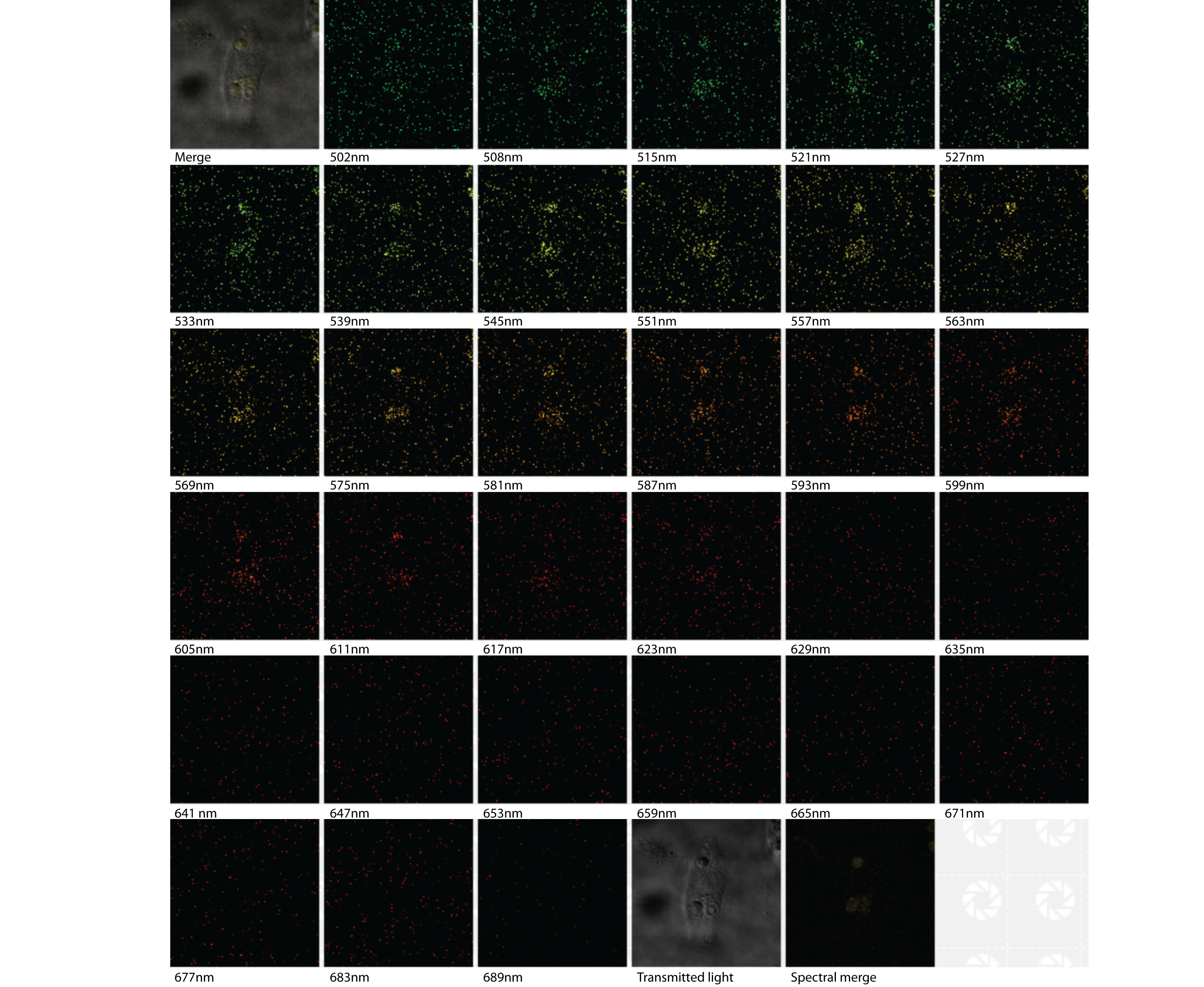

Supplement: Figure S13 — Individual spectral channel images of autofluorescence of intracellular R265 strain. A1R confocal settings used were: 512×512 pixel scan area, 32 channels with 6 ;nm resolution between 500.1 ;nm and 691.3 ;nm, 6 ;mW 488 laser line, 255 spectral detector gain, 110 transmitted light detector gain, 0.11 ;µm/pixel. (TIF) [file pone.0015968.s013.tif]
