# Supplementary material for: Automated Analysis of Cryptococcal Macrophage Parasitism Using GFP-Tagged Cryptococci
Source: PLoS One. 2010 Dec 31;5(12):e15968. doi: 10.1371/journal.pone.0015968 (PMC3013146; doi:10.1371/journal.pone.0015968)
Supplement: Table S1 — Statistical Analysis. P‐values of statistical analysis of results from CFU counts from stress treatments and intracellular virulence assays of GFP‐positive strains compared to parental strains. (DOC) [file pone.0015968.s014.doc]

**Table S1: Statistical Analysis.** P-values of statistical analysis of results from CFU counts from stress treatments and intracellular virulence assays of GFP-positive strains compared to parental strains.

| **Strain**  **Condition** | | **R265** | | | | **H99** |
| --- | --- | --- | --- | --- | --- | --- |
|  |  | **GFP6** | **GFP13** | **GFP14** | **GFP15** | **GFP** |
|  | **25°C** | 0.993 | 0.958 | 0.992 | 1.000 | 0.967 |
|  | **37°C** | 0.998 | 1.000 | 0.970 | 1.000 | 0.875 |
|  | **3% O_2_** | 0.989 | 1.000 | 0.998 | 1.000 | 1.000 |
| **CoCl_2_** | **0.05 mM** | 0.982 | 0.945 | 0.999 | 1.000 | 0.943 |
|  | **0.1 mM** | 1.000 | 0.997 | 1.000 | 0.972 | 0.995 |
|  | **0.3 mM** | 0.999 | 1.000 | 1.000 | 0.999 | 0.979 |
| **H_2_O_2_** | **0.25 mM** | 1.000 | 1.000 | 0.999 | 0.999 | 0.995 |
|  | **0.5 mM** | 0.991 | 1.000 | 1.000 | 1.000 | 0.953 |
|  | **1 mM** | 0.992 | 1.000 | 1.000 | 1.000 | 0.999 |
|  | **5 mM** | 0.154 | 0.963 | 0.361 | 0.185 | 1.000 |
| **NaNO_2_** | **1 mM** | 1.000 | 1.000 | 0.990 | 1.000 | 0.861 |
|  | **5 mM** | 1.000 | 0.999 | 1.000 | 1.000 | 1.000 |
|  | **20 mM** | 1.000 | 0.999 | 0.997 | 1.000 | 0.849 |
| **SDS in DMEM** | **0.005 %** | 0.832 | 1.000 | 1.000 | 1.000 | 1.000 |
|  | **0.01 %** | 1.000 | 1.000 | 1.000 | 0.993 | 1.000 |
|  | **0.05 %** | 1.000 | 1.000 | 0.988 | 1.000 | 1.000 |
| **SDS in YPD** | **0.005 %** | 1.000 | 0.778 | 0.890 | 0.890 | 1.000 |
|  | **0.01 %** | 0.467 | 0.969 | 0.983 | 0.323 | 0.771 |
|  | **0.05 %** | 1.000 | 1.000 | 0.924 | 1.000 | 0.924 |
| **NaCl** | **0.05 mM** | 0.998 | 0.892 | 0.875 | 1.000 | 1.000 |
|  | **0.1 mM** | 0.971 | 0.993 | 1.000 | 1.000 | 1.000 |
|  | **0.3 mM** | 0.986 | 1.000 | 1.000 | 1.000 | 1.000 |
| **Percentage Phagocytosis** | | 0.877 | 0.969 | 0.794 | 0.918 | 0.851 |
| **IPR** | | 0.972 | 0.916 | 1.000 | 0.989 | 0.989 |
| **Occurrence of Expulsion** | | 0.934 | 0.718 | 0.904 | 0.433 | 0.264 |
